# *Acbd7* is essential for preserving hair cell-mediated auditory and vestibular function

**DOI:** 10.1038/s12276-026-01812-1

**Published:** 2026-08-14

**Authors:** Mingxuan Wu, Gaogan Jia, Yanyan Jia, Yikang Huang, Yunjie Li, Yaoqian Liu, Yiyun Lou, Tianshi Sun, Xintong Qiu, Mingyu Xia, Huawei Li, Wenyan Li

**Affiliations:** 1 ENT Institute and Department of Otorhinolaryngology, Eye and ENT Hospital, State Key Laboratory of Medical Neurobiology and MOE Frontiers Center for Brain Science, Fudan University, Shanghai, China; 2 Institutes of Biomedical Sciences, Fudan University, Shanghai, China; 3 NHC Key Laboratory of Hearing Medicine, Fudan University, Shanghai, China; 4 The Institutes of Brain Science and The Collaborative Innovation Center for Brain Science, Fudan University, Shanghai, China; 5 Shanghai Key Laboratory of Gene Editing and Cell Therapy for Rare Diseases, Fudan University, Shanghai, China; 6 School of Life Sciences, Zhejiang Chinese Medical University, Hangzhou, China

**Keywords:** Hair cell, Calcium signalling

## Abstract

Understanding the molecular basis of hair cell function is essential for elucidating inner ear physiology and developing therapies for auditory–vestibular disorders. Here, we identify acyl-CoA binding domain-containing 7 (*Acbd7*) as a hair cell-specific gene critical for sensory maintenance. Single-cell transcriptomics of mouse cochlear organoids revealed *Acbd7* as a top hair cell-enriched transcript, with its spatiotemporal expression confirmed from embryonic development through adulthood in both auditory and vestibular hair cells. *Acbd7*‑deficient mice exhibited pronounced hair cell degeneration, characterized by synaptic defects and diminished calcium currents in inner hair cells and loss of outer hair cells. Transcriptomic and proteomic analyses linked *Acbd7* to the regulation of Ca^2+^ signaling and fatty acid metabolism pathways. Our findings establish *Acbd7* as a critical regulator of Ca^2+^ homeostasis and functional integrity in hair cells, thereby elucidating a key mechanism by which a fatty acid metabolism factor sustains hair cell function and providing potential therapeutic targets for inner ear disorders.

## Acbd7 gene essential for hair cell function revealed

Hearing loss, a significant global health issue, affects millions and often results from damage to cochlear hair cells. This study identifies Acbd7 as a crucial gene for maintaining hair cell function in the auditory and vestibular systems. Researchers used genetic ablation models to demonstrate that Acbd7 is essential for calcium homeostasis and synaptic integrity in hair cells. They used techniques such as single-cell RNA sequencing, immunofluorescence, and electrophysiological recordings to explore the role of Acbd7. Results showed that Acbd7 deficiency leads to progressive hearing loss and outer hair cell degeneration, highlighting its importance in synaptic transmission and hair cell maintenance. The study suggests that Acbd7 could be a therapeutic target for auditory–vestibular disorders. Future research could focus on the potential role of Acbd7 in human hearing and balance, offering insights into novel treatment strategies.

This summary was initially drafted using artificial intelligence, then revised and fact-checked by the author.

## Introduction

Hearing constitutes a fundamental sensory modality for environmental awareness and social communication^1^. According to WHO estimates, more than 430 million individuals worldwide experienced disabling hearing loss in 2021, representing 5.3% of the global population^2^. Sensorineural hearing loss, the predominant form of auditory dysfunction, primarily results from irreversible damage to cochlear hair cells or auditory neural pathways caused by genetic mutations, noise trauma, ototoxic agents, or age-related degeneration^3^. This condition not only severely compromises communication abilities but also elevates risks of anxiety, depression, and cognitive decline, imposing substantial socioeconomic burdens^4,5^.

Cochlear hair cells are the fundamental mechanoreceptors of the auditory system^6^, which functions by converting sound stimuli into electrical signals that the brain processes^7^. Within the cochlea, inner hair cells (IHCs) serve as the primary sensory receptors, transmitting auditory signals through synaptic connections with spiral ganglion neurons (SGNs)^8^, whereas outer hair cells (OHCs) act as mechanical amplifiers to enhance auditory sensitivity and frequency tuning^9^. Analogously, vestibular hair cells — comprising type I and type II subtypes — orchestrate balance perception and form synaptic connections with vestibular ganglion neurons (VGNs)^10,11^, with their dysfunction leading to debilitating equilibrium disturbances and fall-related morbidity^12,13^.

The mechanoelectrical transduction (MET) capacity of hair cells arises from structural specializations, featuring apical stereociliary bundles^14,15^ and basolateral synaptic complexes that interface with SGNs or VGNs^16–18^. Acoustic and positional stimulation induces stereociliary bundle deflection, activating MET channels^19,20^. The cation influx through these MET channels depolarizes the hair cell membrane^21^, triggering voltage‑gated calcium channels at the presynaptic active zone (AZ)^22–24^. The resulting Ca^2+^ influx mediates synaptic vesicle fusion and glutamate release into the hair cell–ganglion neuron synaptic cleft, activating postsynaptic receptors on neuronal terminals^22,25^ and ultimately initiating action potentials^26^. This signaling cascade requires precise regulation of intracellular ion dynamics. The absence of effective therapies for hair cell degeneration highlights the critical demand for discovering molecular targets that preserve hair cell function. Specifically, several key regulators of IHC synaptic function have been identified in the auditory system. Notably, otoferlin (encoded by *Otof*), localized to the presynaptic AZ, orchestrates vesicle cycling in IHCs^27,28^, whereas vesicular glutamate transporter 3 (Vglut3, encoded by *Slc17a8*) mediates glutamate loading into synaptic vesicles^29,30^. The AZ scaffold protein Piccolino establishes the structural framework of cochlear IHC synapses, which is essential for normal auditory processing^31^. In addition, cochlear OHCs are electromotile and have a crucial role in amplifying sound vibrations and refining frequency tuning^32,33^. Mammalian OHC electromotility is mainly mediated by the motor protein Prestin^34,35^.

Previously, we developed mouse cochlear organoids with SGN-innervated functional synapses^36^. Single-cell RNA sequencing of these organoids enabled systematic identification of hair cell-specific genes, revealing *Acbd7* — which encodes an acyl-CoA-binding domain protein — as a candidate regulator of hair cell function. The ACBP gene family demonstrates evolutionary conservation from zebrafish to humans and has critical roles in long-chain fatty acid metabolism, neurogenesis, and neuronal regulation^37^. For example, *Acbd1*, a structurally homologous family member, has been shown to modulate lipid metabolism^38^ and regulate the function of GABA receptors, thereby regulating neuronal proliferation and brain development^39^. Genetic ablation of *Acbd5* induces systemic lipid dysregulation accompanied by progressive cerebellar ataxia and neuropathological alterations in the retina and spinal cord^40^, whereas loss-of-function mutations in *Acbd6* are associated with neurodevelopmental disorders characterized by progressive sensorimotor and cognitive impairments^41^. Notably, *Acbd7* remains understudied, although one study suggested its involvement in hypothalamic neuronal circuits controlling food intake and energy expenditure^42^. Importantly, *Acbd7* has emerged as a potential regulator of inner ear hair cell biology: its expression is detected in neonatal hair cells^43^ and upregulated during hair cell regeneration^44^, implying potential functional roles in hair cell mechanosensory transduction. However, the mechanistic basis and physiological function of *Acbd7* in inner ear development and function remain to be fully elucidated.

In this work, we identified *Acbd7* as a conserved hair cell marker in both the auditory and vestibular systems. Using genetic ablation models, we demonstrate that *Acbd7* is essential for maintaining Ca^2+^ homeostasis and functional integrity in hair cells. This work establishes *Acbd7* as a central coordinator of hair cell neurotransmission and a promising therapeutic target for auditory–vestibular disorders.

## Materials and methods

### Mouse model and genotyping

All mouse strains used in this study, including both males and females, had a C57BL/6 background that carries the *Cdh23 Ahl* variant^45,46^. Mice with targeted deletions or floxed alleles of the *Acbd7* gene (exons 2–4) were purchased from GemPharmatech Co., Ltd. The *Pou4f3*-CreERT2 mouse strain was obtained from the Guoqiang Wan Laboratory at Nanjing University. To conditionally ablate the *Acbd7* gene in hair cells, mice carrying the floxed *Acbd7* allele were crossed with the *Pou4f3*-CreERT2 strain. Tamoxifen was administered to the neonatal mice to induce *Acbd7* gene deletion at postnatal days 2–4 (P2–4). Our study examined male and female animals, and similar findings were observed for both sexes. All the animals were bred in the Fudan University Animal Facility and all animal experiments were approved by the Institutional Animal Care and Use Committee of Fudan University. Genotyping was performed via PCR to confirm the presence of transgenes, with primer sequences listed in Supplementary Table 1.

### Auditory brainstem response testing

Mice were anesthetized with a combined anesthetic at a dosage of 10 μl/g body weight. Each 10 ml of the anesthetic contained 40 mg of Zoletil^®^ 50 (Virbac Corp.) and 100 μl of dexmedetomidine hydrochloride injection (Dexdomitor^®^, Zoetis, Inc.). After anesthesia, the mice were placed in a soundproof chamber on a warming pad to maintain body temperature. The auditory brainstem response (ABR) system (RZ6, Tucker-Davis Technologies (TDT)) was used to measure auditory responses. Short tone-burst stimuli (3 ms duration, 1 ms rise or fall times) were delivered via free field through an MF-1 speaker, which was 10 cm from the vertex at a rate of 20 per second. Auditory function in the mice was assessed by ABR thresholds. The mice were exposed to pure tone pips at various sound frequencies (4, 8, 16, 24, 32 kHz, and click) in the soundproof chamber. For each measured frequency, the sound level started at 90 dB sound pressure level (SPL) and was decreased in 5 dB steps until it reached two levels below the visible threshold. Hearing tests were conducted from 1 month of age (the onset of hearing) to 12 months of age, with all measurements performed by an investigator blinded to the mouse genotype.

### Distortion product otoacoustic emissions measurement

After anesthesia, distortion product otoacoustic emissions (DPOAEs) were measured in mice using the TDT RZ6 real-time processor and BioSigRZ software. Stimulus sounds were generated by a TDT EC1 insert probe (integrated with a speaker and a microphone), with binaural pure-tone stimulation parameters set as follows: frequency ratio f2/f1 = 1.2, the SPL of f1 10 dB higher than that of f2, and target test frequencies for f2 covering 8, 16, 24, and 32 kHz. Acoustic signals from the external auditory canal were synchronously collected by the system, following filtering and noise reduction, and the DPOAE amplitude at the frequency of 2f1−f2 was extracted. A valid DPOAE was defined as an amplitude significantly higher than the noise floor, and the SPL corresponding to the minimum valid response at each frequency was designated as the DPOAE threshold.

### Vestibular behavior testing

Vestibular function was assessed using a comprehensive set of behavioral tests designed to evaluate balance and spatial orientation in the mice. The following tests were included.

#### Open field test

Open field tests, including retropulsion and rotation tests, were conducted to assess locomotor and balance ability. The mice were allowed to roam uninhibited within a 60 cm × 60 cm arena, and the incidences of retropulsion and rotational behaviors were recorded.

#### Anti-gravity test

The anti-gravity test comprised the tail-hanging reflex and air-righting reflex. The tail-hanging reflex was performed by slowly lifting the mouse by the base of its tail to a height of ~10 cm above the horizontal surface and then gently lowering it back to the starting position. Mice with a normal response that stretched their forelimbs toward the ground were scored 0; those with abnormal behavior that showed ventral bending of the body were scored 1. For the air-righting reflex test, mice were placed on their backs at a height of ~30 cm above a soft-landing surface. Upon rapid release, their falling and righting attempts were observed. The time required for the mouse to correct its head position in mid-air was measured; if this was not achieved within 0.2 s, it was noted as a “timeout.” High-speed recordings of both anti-gravity reflex tests were captured using a GoPro Hero 8 camera, at 240 frames per second to ensure detailed documentation of mice movements.

#### Swimming test

Mice were gently immersed in a water bath maintained at a constant temperature of 37 °C, and their behavioral responses were captured. The assessment criteria for their aquatic behavior were as follows: mice exhibiting normal swimming behavior were awarded a score of 0; those displaying abnormal swimming patterns received a score of 1; those that remained stationary and floating earned a score of 2; and those struggling while submerged were given a score of 3.

#### Off-vertical axis rotation and angular vestibulo-ocular reflex tests

Off-vertical axis rotation (OVAR) and angular vestibulo-ocular reflex (aVOR) experiments were conducted using a binocular nystagmography vestibular function testing system (Shenzhen Giant (Ju’an) Technology Co., Ltd, GAT-MVOR943). For the OVAR assessment, the mice were affixed to a platform inclined at a 17° angle from the horizontal, which underwent constant angular velocity rotation at 50°/s. The ocular movements were recorded for over 90 s, and the amplitude of the eye movements was calculated using customized software, providing a comprehensive evaluation of the vestibular responses of the mice. During the aVOR assessment, the mice were securely positioned on a rotating platform using a non-invasive apparatus. In a controlled dark setting, the platform delivered horizontal sinusoidal rotational stimuli with a peak velocity of 40°/s. The experimental protocol encompassed a range of five frequencies: 0.5, 0.8, 1.0, 1.6, and 3.2 kHz each applied to the mice for more than 90 s. Ocular movements were meticulously captured under infrared lighting at 60 frames per second, using a dual camera system. The data were subsequently analyzed using GiantVOR software (v1.0) to determine the gain, which represents the ratio of the ocular response amplitude to the rotational stimulus amplitude.

### Cochlear epithelium harvesting and cryosectioning

After mice were euthanized, the cochleae were extracted and placed in 4% paraformaldehyde (PFA). The apical portion of the cochlear bone was carefully removed, and the round window membrane and the oval window membrane were punctured. The cochleae were then thoroughly perfused with 4% PFA through the round and oval windows and subsequently fixed overnight at 4 °C in 4% PFA. Decalcification was performed in a 10% EDTA solution.

For whole-mount staining, cochlear epithelia were dissected from the cochleae. For cochleae requiring sectioning, samples were sequentially immersed in 15% and 30% sucrose solutions overnight at 4 °C for cryoprotection. Excess moisture was removed by blotting, and the cochleae were infiltrated with optimal cutting temperature compound at 4 °C overnight to ensure thorough penetration. After infiltration, the cochleae were frozen and sectioned on a Leica cryostat, at a thickness of 10 μm. The sections were mounted on adhesion slides (80312-7161-16, CITOTEST) for subsequent immunofluorescence staining or RNA Scope analysis.

### Immunofluorescence and confocal imaging

The samples were blocked and permeabilized in a phosphate-buffered saline solution containing 10% serum and 1% Triton X-100 (Invitrogen^TM^, HFH10) at room temperature for 30 min. After removing the blocking solution, primary antibodies were applied, and the samples were incubated at 4 °C overnight. Following the overnight incubation, unbound primary antibodies were removed by three 15-min washes. The primary antibodies used in this study were listed in Supplementary Table 2. The secondary antibodies were then added, and the samples were incubated at room temperature for 2 h. Then, the samples were washed three times, with each wash lasting 15 min. The samples were subsequently incubated with 4′,6-diamidino-2-phenylindole (1:800, 62248; Thermo Scientific^TM^) for 5 min to stain the nuclei. Finally, the samples were mounted in anti-fade glycerol to prevent fluorescence quenching. Confocal imaging was performed using a Leica SP8 and a Leica STELLARIS 8 confocal microscope, and images were processed using ImageJ software.

Fluorescence images were acquired using identical laser intensity, gain, and exposure parameters across all samples to minimize technical variation. The fluorescence intensity was measured by ImageJ software. For each image, regions of interest were selected, and mean fluorescence intensity was calculated as the average fluorescence intensity of all pixels within the regions of interest.

### RNAscope in situ hybridization assay

The RNAscope^®^ in situ hybridization assay was performed on 10-μm thick cochlear tissue sections. Following deparaffinization and rehydration, tissue sections were pretreated by boiling in RNAscope Target Retrieval Reagent, followed by incubation with RNAscope Protease Plus. RNAscope probes specific to *Acbd7* (Cat. No. 866901-C3, Advanced Cell Diagnostics) were then hybridized to the sections at 40 °C for 2 h. Signal amplification was achieved through the sequential application of RNAscope Amplifier Reagents 1–6, followed by detection with RNAscope 2.5 HD Detection Reagents (red or brown). The sections were counterstained with hematoxylin, dehydrated, cleared, and mounted with a permanent mounting medium. The stained sections were examined and imaged using a brightfield microscope.

### Mouse cochlear organoid culture

Temporal bones containing the cochlea were collected at P0–1 from mice of both sexes, and the cochlear epithelia were meticulously exposed after gently removing the stria vascularis and modiolus. The dissected tissues were then incubated in 0.125% trypsin-EDTA (25200072, Gibco^TM^) for 15 min at 37 °C and rinsed with DMEM/F-12 GluMax medium (10565-018, Gibco) supplemented with 10% fetal bovine serum (A5670701, Gibco) to terminate digestion, followed by rigorous pipetting, and the cells were subsequently filtered through a 40 µm strainer. The cells were resuspended in an expansion medium supplemented with an equal volume of growth factor reduced Matrigel (Corning, 354230). A droplet of 60 μl of the mixture was seeded onto the bottom of a 24-well plate and allowed to solidify into a dome at 37 °C for 45 min before the addition of the expansion medium. The expansion medium was formulated on the basis of DMEM/F-12 GluMax medium (10565-018, Gibco) and supplemented with a cocktail of supplements: 1% N2 supplement (17502001, Gibco), 2% B27 supplement (17504044, Gibco), 2% penicillin–streptomycin (15070063, Gibco), 50 ng/ml epidermal growth factor (EGF) (315-09, PeproTech), 50 ng/ml basic fibroblast growth factor (bFGF) (450-33, PeproTech), 50 ng/ml insulin-like growth factor (IGF) (250-19, PeproTech), and 4 µM CHIR99021 (SML1046, Sigma‒Aldrich). The medium was changed every 3 days to maintain optimal conditions for cell growth. Hair cell differentiation of the organoids was induced by switching to adherent culture and differentiation media supplemented with 5 µM LY411575 (S2714, Selleck).

### Bulk RNA sequencing and analysis

RNA was extracted from cochlear tissues using a RNeasy Micro Kit (Qiagen) and a standard RNA extraction protocol. A PrimeScript^TM^ RT Reagent Kit with gDNA Eraser Kit (TaKaRa) was used to remove genomic DNA according to the manufacturer’s instructions. The quality and quantity of the RNA were assessed before proceeding with library preparation for next-generation sequencing. The RNA-seq libraries were prepared using the SmartSeq2 method and sequenced on the Illumina HiSeq 2500 platform. The data were analyzed to identify differentially expressed genes (DEGs) and clarify the molecular changes associated with *Acbd7* manipulation. The “DESeq2” (v. 1.38.3) package was used to perform DEG analysis, and heatmaps were generated with “Complexheatmap” (v. 2.14.0) to reflect the quantile distribution of the data in the form of a scaled color range.

### Single-cell RNA sequencing and analysis

The mouse cochlear organoid samples used for single-cell sequencing were the same as those used in our previous research^47^. As described in our previous research, organoids differentiated on day 25 were dissociated, and sorting was performed via a MoFlo^®^ SX FACS cytometer (Beckman Coulter). Single-cell transcriptomic profiling of the sorted living single cells was performed using the Single Cell 3’ Reagent v3 kits (10× Genomics), which were then loaded on a 10× Genomics instrument to generate single-cell gel beads. Each bead contains a unique barcode to identify each single cell and a unique molecular identifier to exclude PCR duplicates. Subsequently, the mRNAs were subjected to reverse transcription, cDNA amplification, and library construction based on the Illumina HiSeq 4000 system.

The raw 10× genomics data were converted to FASTQ format and then aligned to the mm10-mouse reference genome to generate count matrix files using CellRanger software (v.7.0.1). For further downstream analysis, the count matrices were processed through a series of R packages (v.4.2.2). Seurat files were generated from the feature count using R package Seurat (v.5.0.2). Low-quality cells with fewer than 200 genes detected, more than 5% mitochondrial percentage, and fewer unique molecular identifier counts than 1,000 were filtered out for all three samples. The filtered matrices were then combined into one Seurat file. The normalization and stabilization of variance of the after-qc Seurat file were performed through an R package, “sctransform” (v.0.4.1), with 3,000 variances identified. The data then went through functions of “RunPCA,” “FindNeighbors,” and “FindClusters” with a resolution of 0.15 for clustering. We used Uniform Manifold Approximation and Projection (UMAP) as the dimension reduction method to analyze the top 30 principal components, outputting 2 dimensions and dividing the data into 8 clusters.

The clustered file was subjected to the function “FindAllMarkers” to identify exclusively expressed markers of every cluster. Two or more known genes among these markers helped identify the cell-type identity of every cluster. The expression levels of the top genes of every cluster were illustrated through heatmaps, using the “DoHeatmap” function of “Seurat” (v.5.0.2) and “vlnplots”, using “Vlnplot“ function.

### Calcium imaging

To quantify hair cell intracellular calcium dynamics after *Acbd7* knockout (KO), adherent cultured mouse cochlear organoids derived from wild-type (WT) and *Acbd7* KO mice were seeded onto confocal dishes before incubation with 5 µM Fura-2 AM working solution. During incubation, 0.02% Pluronic F-127 was added to the Fura-2 AM working solution to enhance membrane permeability. The organoids were incubated with the working solution at 37 °C for 15 min. After incubation, the organoids were gently washed with PBS to remove excess Fura-2 solution. A Leica SP8 confocal microscope with the appropriate excitation and emission wavelengths (340 nm and 380 nm for excitation and 530 nm for emission) was used for signal acquisition, and 50 μM KCl or L-glutamate was used as a stimulant.

### Electrophysiological recordings

After mice were euthanized, the cochleae were promptly extracted and immersed in basic culture medium. The bony structure of the apical turn was carefully removed, and the apical cochlear sensory epithelium was isolated. The vestibular membrane and the tectorial membrane were removed, and the tissue was then placed in a recording chamber containing the external solution mounted on an inverted microscope. Recordings were performed using conventional whole-cell patch-clamp techniques at room temperature (20–25 °C). IHCs were visualized with a 60× water-immersion objective on an Olympus microscope. Currents were recorded using an EPC10 dual-channel amplifier (HEKA Electronics, Lambrecht/Pfalz, Germany) controlled by Patchmaster software (HEKA Electronics). Recording micropipettes were made from borosilicate glass tubes (BF150-86-10, Sutter Instruments, Novato, CA, USA) using a two-stage vertical pipette puller (Narishige, Tokyo, Japan) to tip diameters of ∼1 µm. After establishing a high-resistance seal (>1 GΩ), whole-cell access was obtained by gentle suction.

#### Whole-cell calcium currents and membrane capacitance recordings

To record the whole-cell calcium currents and membrane capacitance of IHCs, the apical turn of cochlea was bathed in an oxygenated extracellular solution containing (in millimolar) 125 NaCl, 5.8 KCl, 5 CaCl_2_, 0.9 MgCl_2_, 10 HEPES, 5.6 D-glucose, 0.7 NaH_2_PO4·H_2_O, and 2 Na-pyruvate adjusted to pH 7.2 with NaOH, 300 mOsm/l with NaCl. Patch pipettes coated with dental wax had a resistance of 5–6 MΩ full of internal solutions containing (in millimolar): 135 Cs-methane sulfonate, 10 CsCl, 10 TEA-Cl, 10 HEPES, 3 Mg-ATP, 2 EGTA, and 0.5 Na-GTP, pH 7.2 adjusted with NaOH, 290 mOsm/l. Cells were held at −90 mV under voltage-clamp conditions. The liquid junction potential (−10 mV) was corrected offline by adjusting all membrane potentials accordingly. Ca^2+^ currents were recorded using a 300 ms voltage ramp protocol ranging from −90 mV to +70 mV, and the maximal current amplitude (*I*_Ca_) was extracted for analysis. Membrane capacitance (*C*_m_) was measured using the “Sine + DC” approach implemented in Patchmaster under voltage-clamp conditions, with a software-based lock-in amplifier. After establishing a high-resistance seal, hair cells were held at −90 mV and stimulated with a 1 kHz sinusoidal command (25 mV amplitude) to monitor *C*_m_. Depolarization-evoked changes in capacitance were calculated as Δ*C*_m_ = *C*_m_(response) − *C*_m_(baseline) and served as a quantitative measure of cumulative synaptic vesicle exocytosis. To assess release kinetics, Δ*C*_m_ was measured in response to depolarizing pulses of increasing durations (2, 5, 10, 20, 50, 100, 200, 500, and 1,000 ms). The resulting Δ*C*_m_–time relationships from individual cells were fitted using a composite model consisting of a single exponential component representing release from the readily releasable pool (RRP; characterized by *C*_m_, RRP, and τ_RRP) and a linear component reflecting sustained release rate (SRR) (*R*_sustained_, SRR):

$${\rm\bigtriangleup }{{\rm{C}}}_{{\rm{m}}}(t)={C}_{{\rm{m}},\mathrm{RRP}}\cdot +(1-\exp (\frac{{\rm{t}}}{{\tau }_{\mathrm{RRP}}}))+{{\rm{R}}}_{\mathrm{sustained}}\cdot {\rm{t}}$$

The number of released synaptic vesicles was estimated from Δ*C*_m_ using a conversion factor of 37 aF per vesicle^48,49^.

#### MET recording

The basilar membrane containing hair cells was acutely dissected from P30 mice and used for electrophysiological recordings within 1 h. Recordings were performed in a standard artificial perilymph containing (in millimolar): 137 NaCl, 5.8 KCl, 1.3 CaCl_2_, 0.9 MgCl_2_, 0.7 NaH_2_PO_4_, 10 HEPES, and 5.6 D-glucose, adjusted to pH 7.4 and 300 mOsm. Patch pipettes were fabricated from borosilicate glass and subsequently fire-polished (MF-830, Narishige) to a resistance of 4–6 MΩ. The internal solution comprised (in millimolar): 140 CsCl, 0.1 EGTA, 10 HEPES, 2 Mg-ATP, 1 MgCl_2_, and 0.3 Na-ATP, titrated to pH 7.4 and 295 mOsm. Mechanotransduction (MET) currents were recorded in whole-cell voltage-clamp mode at a holding potential of –80 mV using an EPC10 dual-channel amplifier operated with Patchmaster software. Signals were filtered at 2 kHz and sampled at 200 kHz. Mechanical stimulation of the hair bundle was delivered by a fluid jet driven by a 40 Hz sinusoidal command applied to a 27 mm piezoelectric disc and amplified by a custom piezo driver. The stimulus pipette (tip diameter 3–5 μm) was positioned 5–10 µm from the bundle to elicit saturating MET responses.

### Electron microscopy

For scanning electron microscopy (SEM), tissue samples were fixed in 2.5% glutaraldehyde in 0.1 M phosphate buffer (pH 7.4) at 4 °C for 2 h. After fixation, the samples were rinsed in phosphate buffer and post-fixed in 1% osmium tetroxide for 1 h at room temperature. The samples were then dehydrated through a graded series of ethanol (30%, 50%, 70%, 90%, and 100%), followed by critical point drying with CO_2_. Once dried, the samples were mounted on aluminum stubs and coated with a thin layer of gold using a sputter coater. Imaging was performed using a SEM (ZEISS GeminiSEM 300), and images were captured at various magnifications to analyze surface morphology.

For transmission electron microscopic analysis of IHCs, sample preparation steps were similar to those used for SEM, except that samples were subsequently embedded in an epoxy resin monomer (90529-77-4, SPI Supplies). Ultrathin sections (70 nm) were cut using an ultramicrotome (Leica EM UC6) and collected on copper grids. Imaging was performed using a TEM (Nippon Tekno JEOL-1230), and images were captured at various magnifications to examine cellular ultrastructure.

### Statistical analysis

Both cochleae from each mouse were allocated to different experimental assays. For immunofluorescent staining quantification, a sample size of *n* ≥ 6 mice was ensured for each readout. One cochlea from each mouse was used for a panel of antibody staining, whereas the contralateral cochlea was allocated to other assays. The same strategy was applied to the vestibular organ. All samples within each quantification group were obtained from independent mice. For organoid experiments, six or more samples were analyzed, and all assays were performed in three independent experimental runs. For electrophysiological recordings, at least 10 cells collected from more than three mice were analyzed per group.

The unpaired two‑tailed Student’s *t* test was applied for comparisons between two groups, whereas one‑way or two‑way analysis of variance followed by Tukey’s post hoc test was used for multiple‑group analyses. *P*-values <0.05 were considered significant: ****P < 0.0001, ***P ≤ 0.001, **P ≤ 0.01, and *P ≤ 0.05. The data are presented as the means ± SEM. All data were analyzed using GraphPad Prism software (version 10.0).

## Results

### Dynamic hair cell-specific expression pattern of *Acbd7* in the mouse inner ear

**Fig. 1:**
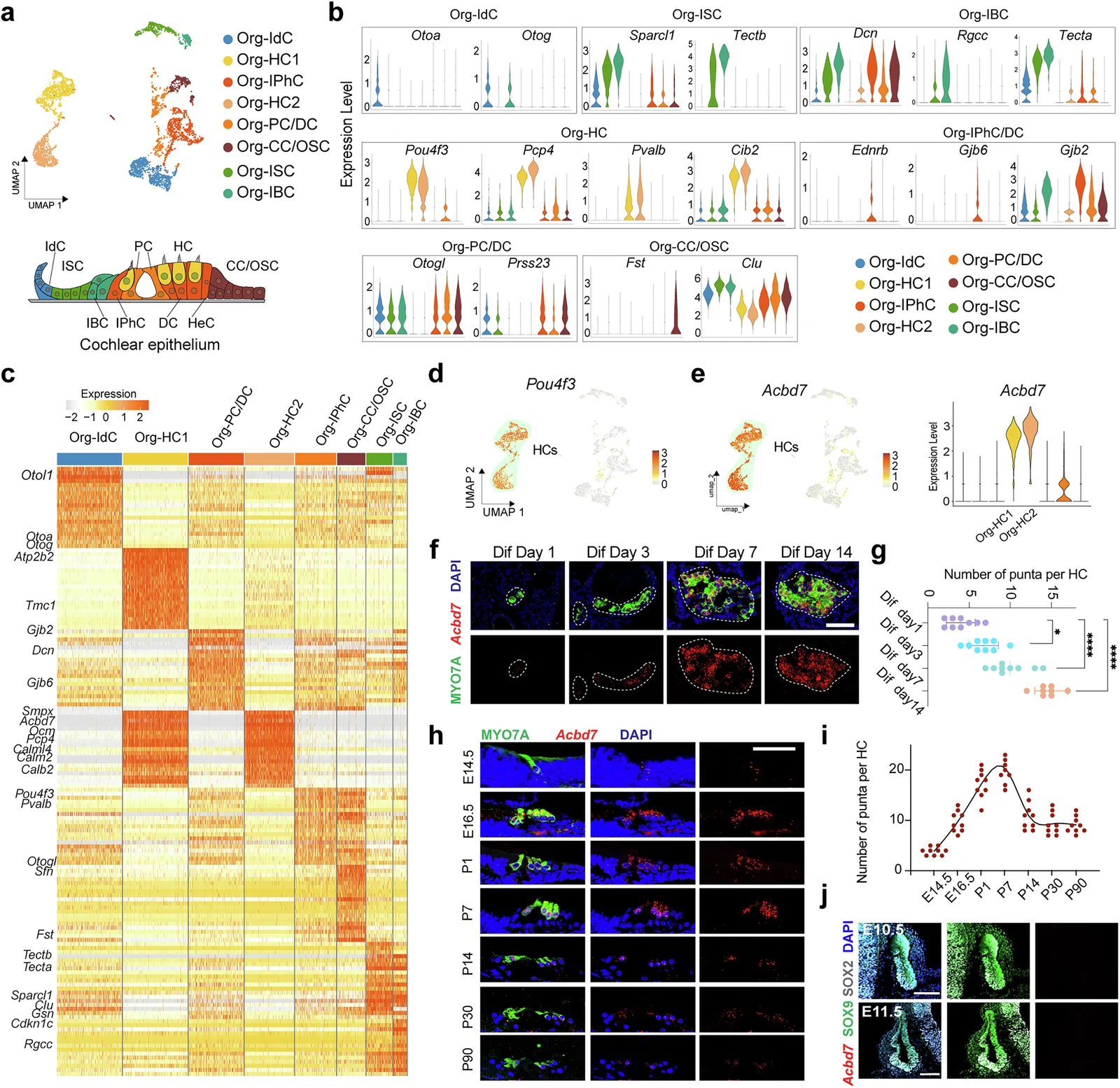
Single-cell transcriptome analysis of mouse cochlear organoids reveals hair cell-specific *Acbd7* expression. **a**, UMAP plot depicting cell-type identity of mouse cochlear organoids (schematic of cochlear epithelium is shown). **b**, Marker gene expression analysis of mouse cochlear organoid cell types. **c**, Heatmap showing the top 25 genes of different mouse cochlear organoid cell types. **d**, Feature plot of hair cell marker *Pou4f3*. **e**, Feature and violin plots showing specific *Acbd7* expression in hair cells. **f**, Dynamic expression of *Acbd7* (red) during mouse organoid hair cell maturation. Hair cells are labeled with MYO7A (green). Scale bar, 50 μm. **g**, Quantification of *Acbd7* expression in mouse organoid hair cells (*n* = 9 organoids from three independent experiments). **h**, Expression pattern of *Acbd7* (red) in developing cochlea from the middle turn. Hair cells are labeled with MYO7A (green). Scale bar, 50 μm. **i**, Quantification of *Acbd7* (red) expression in developing cochlea (*n* = 9 mice per group). Hair cells are labeled by MYO7A staining (green). **j**, No *Acbd7* expression (red) was detected in the otic vesicle before hair cell emergence. Otic progenitors are labeled with SOX9 (green) and SOX2 (gray). Scale bar, 50 μm. One-way analysis of variance and Tukey’s multiple comparison test were performed for part **g**. Data are presented as means ± SEMs. ****P < 0.0001, *P < 0.05. CC, Claudius cell; DAPI, 4′,6-diamidino-2-phenylindole; DC, Deiters’ cell; HC, hair cell; IBC, inner border cell; IdC, interdental cell; IPhC, inner phalangeal cell; ISC, inner sulcus cell; OSC, outer sulcus cell; PC, pillar cell; UMAP, Uniform Manifold Approximation and Projection.

Through single-cell RNA sequencing of innervated mouse cochlear organoids^36^, we comprehensively characterized the transcriptional profiles of cochlear epithelial cell populations. Unsupervised clustering and UMAP visualization delineated eight distinct clusters, each exhibiting unique gene expression signatures that were validated using established markers^43,50^ (Fig. 1a,b). The cellular composition of these terminally differentiated mouse cochlear organoids closely recapitulated the native cochlear epithelium, encompassing cochlear hair cells and supporting cells (SCs), as well as non-sensory components (medial and lateral epithelial cells). Medial epithelial cells, such as interdental cells (identified by *Otoa* and *Otog*), inner sulcus cells (identified by *Sparcl1* and *Tectb*), and inner border cells (identified by *Dcn*, *Rgcc*, and *Tecta*), were annotated. Lateral Claudius cells and outer sulcus cells were distinctly identified by *Fst* and *Clu*. Organoid hair cells were definitively characterized by their expression of *Pou4f3*, *Pvalb*, *Pcp4*, and *Cib2* (Fig. 1c,d and Supplementary Fig. 1a). In addition, we identified two distinct SC subtypes. *Ednrb*, *Gjb6*, and *Gjb2* indicated the identity of inner phalangeal cells, whereas another SC cluster represented pillar cells and Deiters’ cells based on *Prss23* and *Otogl* expression (Fig. 1b).

Comparative analysis (Fig. 1c) of cluster-enriched genes revealed *Acbd7* as one of the top three hair cell-specific transcripts, with particularly robust expression in hair cells (Fig. 1d,e). To validate the expression of *Acbd7* in hair cells, we performed RNAscope in situ hybridization and confirmed that *Acbd7* is predominantly restricted to hair cells and gradually upregulated during mouse cochlear organoid maturation (Fig. 1f,g). Notably, the *Acbd7* transcript signal was restricted to hair cells, and no signal was detected in other regions, including the SGN region (Supplementary Fig. 1b). *Acbd7* was first detected on embryonic day (E)14.5 in the cochlea and was specifically expressed in hair cells from the embryonic to the neonatal stage (Supplementary Fig. 1c,d). The *Acbd7* mRNA signals exhibited progressive upregulation from E14.5 to postnatal day 7 (P7) and persisted into adulthood (Fig. 1h,i). No expression was detected in the prosensory epithelium before hair cell emergence (E10.5–E11.5; Fig. 1j). Furthermore, within the embryonic vestibular epithelium, *Acbd7* was specifically expressed in sensory hair cells, suggesting conserved functional importance across inner ear sensory systems (Supplementary Fig. 1e). To determine the subcellular localization of ACBD7 protein, we generated an *Acbd7*-overexpressing AAV (AAV2/PHP.eB-CMV-*Acbd7*-EGFP-3×Flag-WPRE-pA) and observed strong ACBD7 expression in the hair cell cytoplasm (Supplementary Fig. 1f). Together, these findings establish *Acbd7* as a dynamically regulated, hair cell-specific gene with a potential critical role in both auditory and vestibular function.

### *Acbd7* KO mice exhibit progressive hearing loss and OHC degeneration

**Fig. 2:**
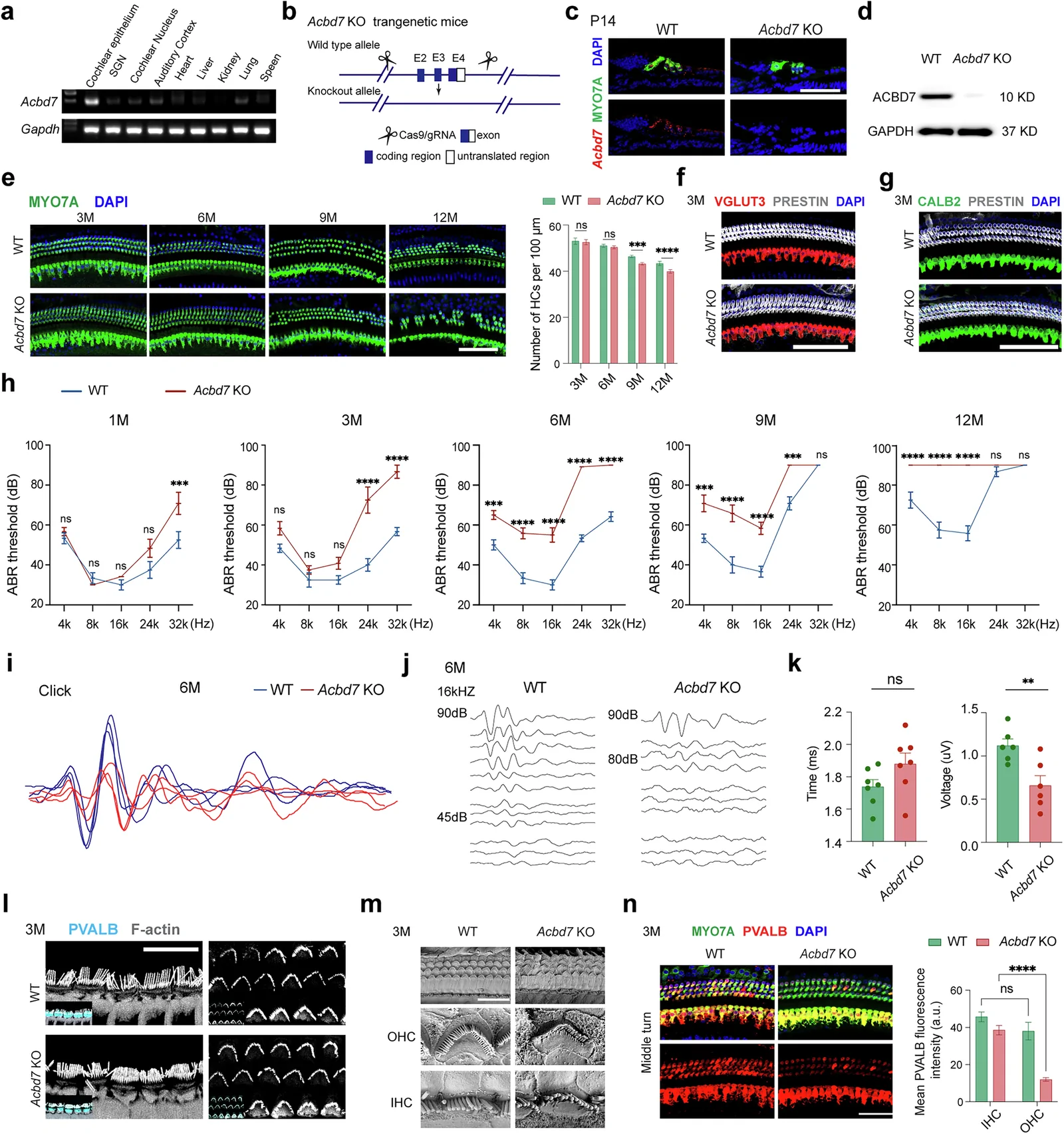
*Acbd7* KO mice exhibit progressive hearing loss and OHC degeneration. **a**, Tissue‑specific expression of *Acbd7* detected by PCR. **b**, Schematic of generation of *Acbd7* KO mouse line. **c**, RNAscope showing *Acbd7* (red) expression in the cochlea from WT and *Acbd7* KO mice. Hair cells are labeled with MYO7A (green). Images were acquired from the middle turn of the cochlea. Scale bar, 50 μm. **d**, Representative western blots showing ACBD7 protein expression in the cochlea. **e**, Representative confocal images showing MYO7A‑labeled hair cells (green) in the cochlea from WT and *Acbd7* KO mice (middle turn of the cochlea). Scale bar, 50 μm. Quantification of MYO7A+ hair cell number is shown on the right (*n* = 12 mice per group). **f**,**g**, Immunofluorescence staining of IHC and OHC markers at 3 months of age: IHCs labeled with VGLUT3 (red) and CALB2 (green); OHCs labeled with PRESTIN (gray). Images were obtained from the middle turn of the cochlea. Scale bar, 50 μm. **h**, ABR thresholds measurement of WT and *Acbd7* KO mice (*n* = 6 mice per group). **i**, Representative images of ABR click wave of 6-month-old WT and *Acbd7* KO mice. **j**, Representative images of ABR wave at 16 kHz of 6-month-old WT and *Acbd7* KO mice. **k**, ABR wave I latency and amplitude of 6-month-old WT and *Acbd7* KO mice (*n* = 6 mice per group). **l**, High-resolution confocal imaging showing F-actin-labeled hair bundles (gray) of 3-month-old WT and *Acbd7* KO. Hair cells are labeled with PVALB staining (cyan). Images were acquired from the middle turn of the cochlea. Scale bar, 20 μm. **m**, Scanning electron microscope showing stereociliary structures in 3-month-old WT and *Acbd7* KO hair cells (middle turn of the cochlea). Scale bar, 20 μm. **n**, Immunofluorescence staining of PVALB (red) and MYO7A (green) in cochlear hair cells from 3-month-old WT and *Acbd7* KO mice. Images were acquired from the middle turn of the cochlea. Scale bar, 50 μm. Quantification of mean PVALB fluorescence intensity in IHCs and OHCs is shown on the right (*n* = 6 mice per group). Unpaired two-tailed Student’s *t* tests were performed for part **k**. Two-way analysis of variance followed by Tukey’s multiple comparisons test was performed for parts **e**, **h**, and **n**. Data are presented as means ± SEMs. ****P < 0.0001, ***P < 0.001, **P < 0.01, *P < 0.05. ABR, auditory brainstem response; a.u., arbitrary unit; DAPI, 4′,6-diamidino-2-phenylindole; gRNA, guide RNA; HC, hair cell; IHC, inner hair cell; KO, knockout; ns, not significant; OHC, outer hair cell; SGN, spiral ganglion neuron; WT, wild-type.

Systemic expression analysis of *Acbd7* in P7 mice by reverse transcription-PCR showed predominant expression in the cochlea, with minimal levels detected in both the central auditory pathway (SGNs, cochlear nucleus, and auditory cortex) and peripheral organs (heart, liver, kidney, lung, and spleen) (Fig. 2a). This tissue-restricted expression pattern of *Acbd7* implied a potentially specialized function in cochlear biology. To investigate this hypothesis, we generated *Acbd7* KO mice using CRISPR/Cas9-mediated genome editing (Fig. 2b). The targeted deletion of exons 2–4 resulted in complete ablation of both *Acbd7* mRNA and protein expression (Fig. 2c,d). Longitudinal monitoring over a 12-month period demonstrated progressive cochlear OHC loss in *Acbd7*-deficient mice compared with WT mice (Fig. 2e and Supplementary Fig. 2a,b). Quantitative analysis of key molecular markers — including VGLUT3, Otoferlin, and CALB2 in IHCs and PRESTIN in OHCs — revealed stable expression in *Acbd7* KO animals compared with WT mice (Fig. 2f,g and Supplementary Fig. 2c–e). These results collectively demonstrate that although *Acbd7* is essential for the long-term maintenance of hair cell integrity, it does not affect the molecular identity of hair cells.

ABR measurements revealed significantly elevated thresholds at high frequencies (32 kHz) as early as 1 month of age, with progressive hearing loss that extended across all frequencies and culminated in complete deafness by 12 months in *Acbd7* KO mice (Fig. 2h). Furthermore, the auditory deficits were associated with reduced wave I amplitudes and prolonged latencies (Fig. 2i–k), implying impaired synaptic transmission at the IHC-auditory nerve fiber synapse. Additionally, DPOAE measurements revealed elevated thresholds from 3 months of age, indicating compromised OHC function in mutants (Supplementary Fig. 2f). Together, these findings demonstrate that the hearing loss in *Acbd7* KO mice arises from functional deficits in both IHCs and OHCs. To investigate potential structural correlates, we performed high-resolution imaging using stimulated emission depletion fluorescence microscopy and SEM. Both IHC and OHC stereocilia in homozygous *Acbd7* KO mice maintained normal morphology at 3 months of age, despite the presence of hearing loss at this stage (Fig. 2l,m and Supplementary Fig. 2g). Immunofluorescence staining revealed decreased expression of α-parvalbumin (PVALB), a calcium-binding protein involved in calcium buffering and signaling, in *Acbd7* KO mouse hair cells, with OHCs showing a particularly pronounced reduction (Fig. 2n). The decrease exhibited a base-to-apex gradient along the cochlear tonotopic axis, consistent with the predominantly high-frequency hearing loss observed in *Acbd7* KO mice (Supplementary Fig. 2h,i). By 6 months of age, PVALB expression was further reduced across all cochlear turns (Supplementary Fig. 2j,k), correlating with the progression to all-frequency hearing loss. Collectively, these results suggest that *Acbd7* deficiency leads to progressive hearing impairment and OHC degeneration.

### *Acbd7* maintains IHC synaptic integrity and regulates synaptic transmission

**Fig. 3:**
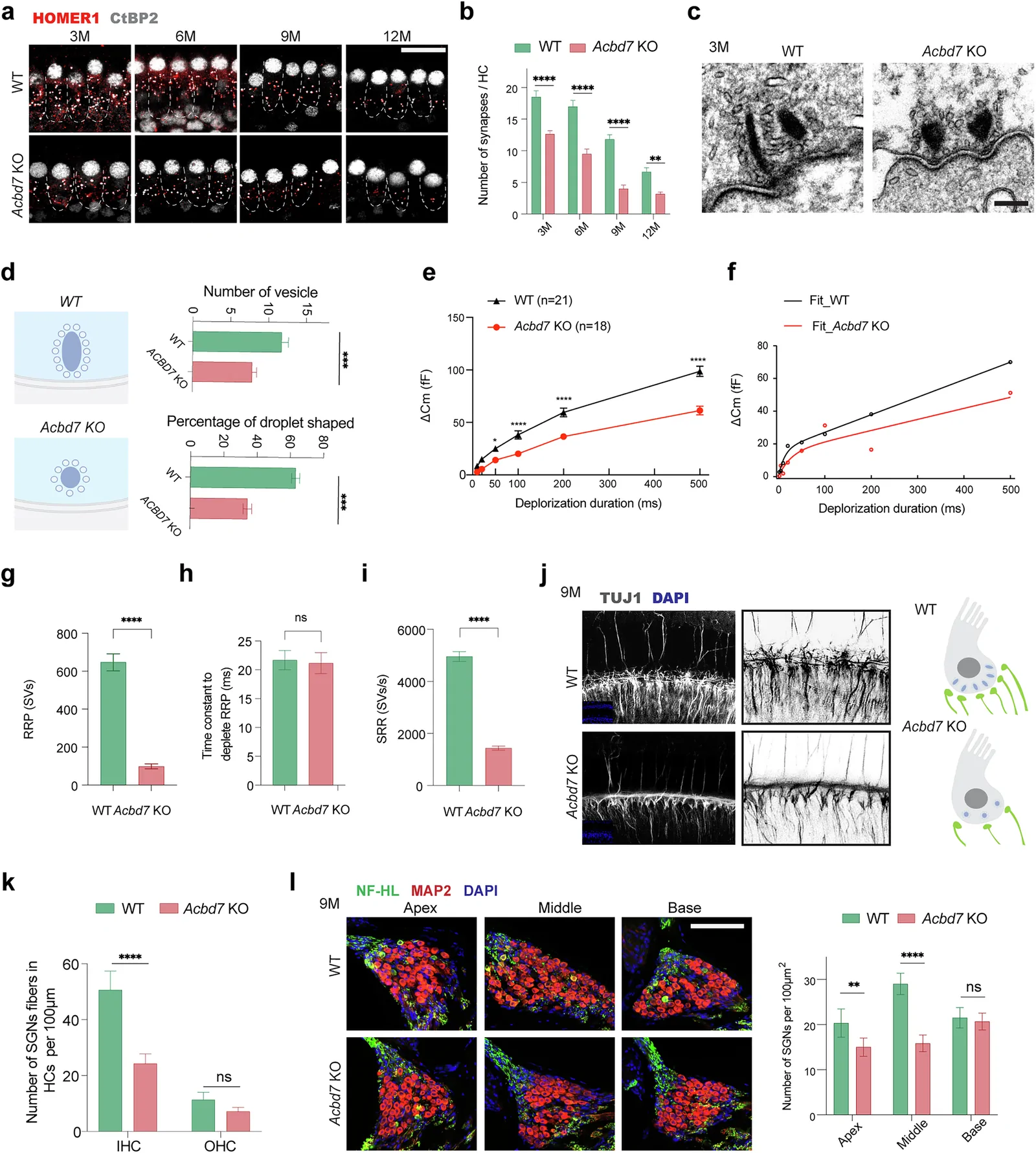
*Acbd7* KO impairs IHC ribbon synapses and SGNs in the cochlea. **a**, Age-dependent impairment of hair cell synapses in *Acbd7* KO mice. Presynaptic components were labeled by CtBP2 (gray) and postsynaptic elements were marked by HOMER1 (red) (middle turn of the cochlea). Scale bar, 20 μm. **b**, Quantification of hair cell synapse showing the decreased number of synapses in IHCs of *Acbd7* KO mice (*n* = 6 mice per group). **c**, Transmission electron microscopy showing morphological changes in IHC ribbons from WT and *Acbd7* KO mice at 3 months of age from the middle turn of the cochlea. Scale bar, 100 nm. **d**, Representative graph and quantification showing the number of synaptic vesicles and percentage of droplet-shaped ribbons in IHCs (*n* = 6 mice per group). **e**, Δ*C*_m_ values measured from 3-month-old wild-type (*n* = 21 cells from 3 mice) and *Acbd7* KO mouse (*n* = 18 cells from 3 mice) IHCs from the apical turn region. **f**, Typical curves obtained by fitting the plots of Δ*C*_m_ against stimulus duration for a pair of WT (black) and *Acbd7* KO mouse hair cells (red). Three parameters of exocytosis were extracted, including time constant (*τ*) to release RRP (part **g**), deplete RRP (part **h**), and sustained release rate (SRR) (part **i**) in wild-type (*n* = 15 cells from 3 mice) and *Acbd7* KO mouse (*n* = 15 cells from 3 mice) hair cells. **j**, TUJ1 staining (gray) showing SGN neuronal fibers in 9-month-old WT and *Acbd7* KO mice (*n* = 6 mice per group), imaged from the middle turn of the cochlea. Scale bar, 50 μm. **k**, Quantification of neuronal fibers in 9-month-old WT and *Acbd7* KO mice cochlea (*n* = 6 mice per group). **l**, Representative images showing the decreased number of SGNs in *Acbd7* KO mice. Neuronal fibers were labeled by NF (green) and SGN labeled by MAP2 (red). Scale bar, 50 μm. Quantification of MAP2+ SGN number is shown on the right (*n* = 6 mice per group). Unpaired Student’s *t* tests (two-tailed) were performed for parts **d**–**i**. Two-way analysis of variance followed by Tukey’s multiple comparisons test was performed for parts **b**, **k**, and **l**. Data are presented as means ± SEMs. ****P < 0.0001, ***P < 0.001, **P < 0.01, *P < 0.05, n.s., not significant. DAPI, 4′,6-diamidino-2-phenylindole; HC, hair cell; IHC, inner hair cell; KO, knockout; OHC, outer hair cell; RRP, readily releasable pool; SGN, spiral ganglion neuron; WT, wild-type.

Given the intact stereociliary morphology in *Acbd7*-deficient mice, we examined whether synaptic defects contribute to the observed auditory dysfunction. Immunofluorescence analysis revealed a progressive reduction in CtBP2/HOMER1-positive synaptic pairs in IHCs of *Acbd7*-deficient mice from 3 to 12 months of age (Fig. 3a,b). Complementary ultrastructural analysis by TEM revealed notable ribbon synapse pathology in 3-month-old *Acbd7* KO mice compared with WT controls. Although WT IHC synapses exhibited the characteristic electron-dense, droplet-shaped presynaptic ribbons surrounded by orderly arranged synaptic vesicles, *Acbd7*-deficient synapses showed marked atrophy of this synaptic architecture (Fig. 3c,d). *Acbd7*-deficient synapses exhibited structural degeneration, manifesting as dissolution of the electron-dense ribbon core and pronounced depletion of presynaptic vesicles.

Whole-cell patch-clamp recordings of IHCs revealed pronounced deficits in synaptic transmission in *Acbd7*-deficient mice (Supplementary Fig. 3a). Membrane capacitance measurements indicated progressively suppressed vesicle release from 1 to 3 months of age (Fig. 3e and Supplementary Fig. 3b). Quantification of vesicle pool dynamics in mature (3‑month‑old) IHCs showed that the RRP was severely reduced in *Acbd7* KO IHCs (98.67 ± 13.31 vesicles versus 646.7 ± 45.22 in WT), although its depletion kinetics remained normal (Fig. 3f–h). By contrast, the SRR was markedly impaired (1436 ± 79.36 SV/s in *Acbd7* KO versus 4957 ± 190.5 SV/s in WT), indicating defective vesicle replenishment (Fig. 3i). These results demonstrate that *Acbd7* deficiency primarily disrupts the maintenance and refilling of synaptic vesicle pools. Voltage-clamp analysis demonstrated reduced calcium channel function, with *Acbd7* KO IHCs exhibiting maximal Ca^2+^ currents of only 100 pA compared with 250 pA in WT controls at 1 month of age (Supplementary Fig. 3c). MET currents of IHCs were comparable between *Acbd7* KO and WT mice (Supplementary Fig. 3d,e), confirming preserved MET activity. Together, these findings demonstrate that *Acbd7* regulates synaptic vesicle pool maintenance and replenishment without affecting mechanosensory function.

Given that synaptic abnormalities in hair cells can perturb spiral SGN firing patterns and disrupt SGN subtype specification^51,52^, we investigated whether *Acbd7* deficiency-induced synaptic loss between IHCs and SGNs leads to SGN degeneration. We observed pronounced neurofilament retraction in *Acbd7* KO mice, particularly within the IHC innervation zone (Fig. 3j). Quantitative analysis confirmed a significant reduction in neurofilament density associated with IHCs in *Acbd7* KO mice compared with WT controls (Fig. 3k). Furthermore, examination of SGN somata revealed substantial neuronal loss in both apical and middle cochlear turns of *Acbd7* KO mice by 9 months of age (Fig. 3l). These findings collectively demonstrate that *Acbd7* has a crucial role in maintaining IHC–SGN synaptic integrity and preserving synaptic transmission function, both of which are essential for normal auditory function.

### *Acbd7* KO mice exhibit abnormal vestibular function

**Fig. 4:**
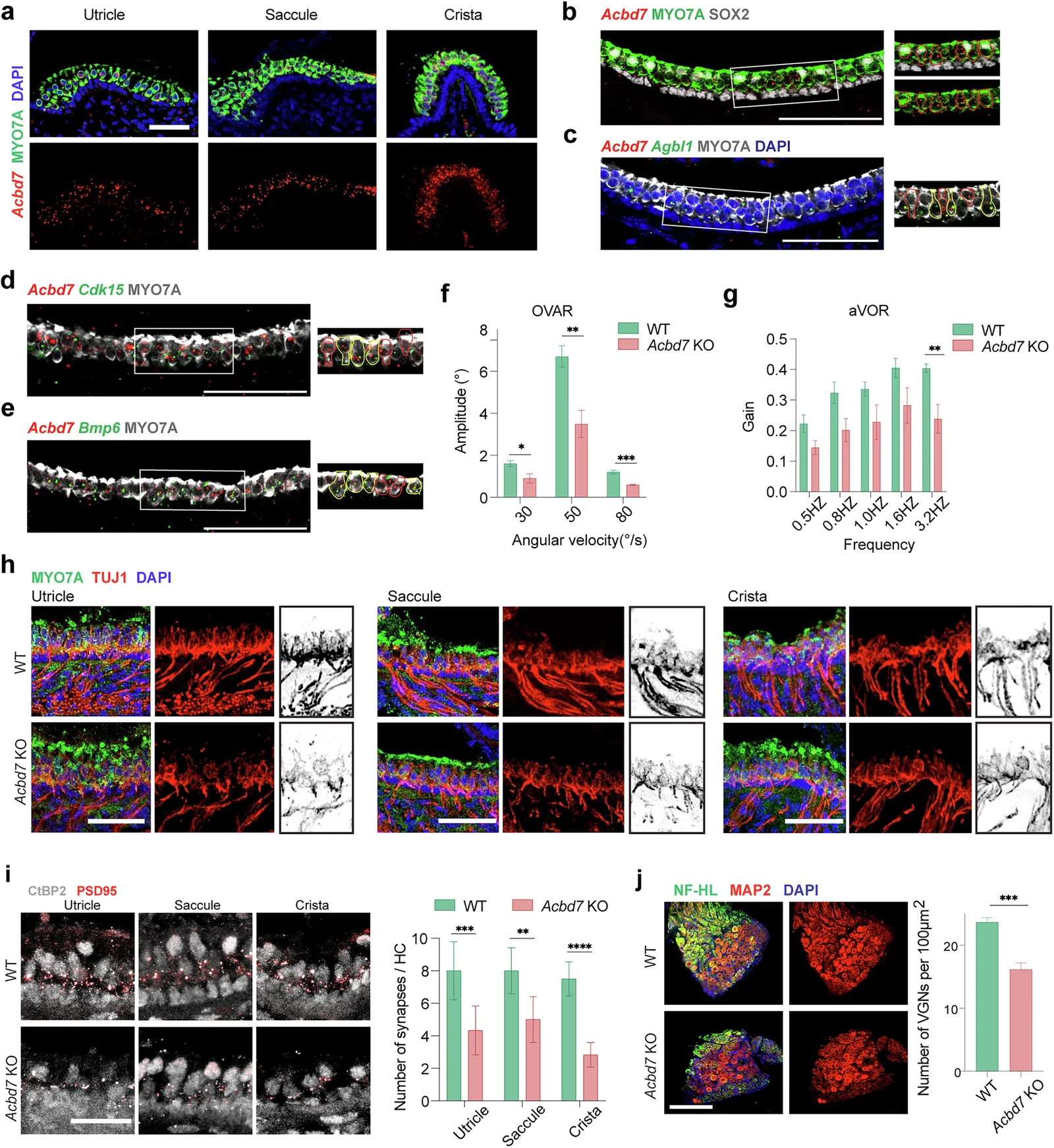
*Acbd7* KO mice exhibit abnormal vestibular function and hair cell synapses. **a**, RNAscope showing *Acbd7* expression (red) in vestibular hair cells (labeled with MYO7A, green) from 1-month-old mice. Scale bar, 50 μm. **b**, RNAscope showing *Acbd7* expression (red) in both vestibular type I and type II hair cells from 3-month-old mice. Type I vestibular hair cells are labeled with MYO7A (green) and type II vestibular hair cells labeled with MYO7A (green) and SOX2 (gray). Scale bar, 50 μm. **c**–**e**, Co‑expression of *Acbd7* (red) with subtype‑specific markers in vestibular hair cells from 3‑month‑old mice. Hair cells are labeled with MYO7A (gray). Red circles indicate *Acbd7*+ cells; yellow circles mark cells double‑positive for *Acbd7* and the subtype marker. **c**, *Acbd7* (red) in *Agbl1*+ (green) extrastriolar type I hair cells. Scale bar, 50 μm. **d**, *Acbd7* (red) in *Cdk15*+ extrastriolar type II hair cells. Scale bar, 50 μm. **e**, *Acbd7* expression in *Bmp6*+ extrastriolar type II hair cells. Scale bar, 50 μm. **f**,**g**, Graphs showing the results of the OVAR and aVOR tests of 9-month-old WT and *Acbd7* KO mice (*n* = 8 mice per group). **h**, *Acbd7* KO leads to decreases in hair cell–neuron innervation of vestibular organs at 9-month-old. Hair cells were stained with MYO7A (green) and VGN (vestibular ganglion neuron) terminals were stained with TUJ1 (red). Scale bar, 50 μm. **i**, Representative images showing the decreased number of synapses in *Acbd7* KO vestibular hair cells at 9 months. Presynaptic components were marked by CtBP2 (gray), and postsynaptic elements were marked by PSD95 (red). Scale bar, 20 μm (*n* = 6 mice per group). **j**, Representative images showing the decreased number of VGNs in 9-month-old *Acbd7* KO mice. Neuronal fibers labeled by NF (green) and VGN somata were labeled by MAP2 (red). Scale bar, 50 μm (*n* = 6 mice per group). Unpaired Student’s *t* tests (two-tailed) were performed for part **j**. Two-way analysis of variance followed by Tukey’s multiple comparisons test was performed for parts **f**, **g**, and **i**. Data are presented as means ± SEMs. ****P < 0.0001, ***P < 0.001, **P < 0.01, *P < 0.05. aVOR, angular vestibulo-ocular reflex; DAPI, 4′,6-diamidino-2-phenylindole; HC, hair cell; KO, knockout; OVAR, off-vertical axis rotation; WT, wild-type.

Given the hair cell-specific expression pattern of *Acbd7* in the cochlea, we first characterized its distribution in vestibular organs. RNAscope in situ hybridization analysis revealed broad *Acbd7* expression in vestibular hair cells of adult mice, including those within the utricle, saccule, and crista ampullaris (Fig. 4a). *Acbd7* was detected in both MYO7A+;SOX2+ and MYO7A+;SOX2− hair cells, confirming its presence in both type I and type II vestibular hair cells (Fig. 4b). The vestibular organs contain four molecularly distinct hair cell subtypes defined by *Agbl1*, *Kcnj16*, *Cdk15*, and *Bmp6* expression patterns^53^. RNAscope in situ hybridization analysis confirmed that *Acbd7* is expressed across all subtypes, showing colocalization with *Agbl1*, *Cdk15*, and *Bmp6* (Fig. 4c–e). Co‑expression with *Kcnj16* was not assessed, as the corresponding probes share the same detection channel. Collectively, these results demonstrate that *Acbd7* is expressed broadly across all vestibular hair cell subtypes without preferential distribution.

To assess vestibular function, we initially performed behavioral tests in 9-month-old *Acbd7* KO mice. Surprisingly, these animals showed no overt vestibular deficits in standard assays including gait analysis (retropulsion and rotation tests) and anti-gravity reflex assessments (tail-hang, swimming, and air-righting tests) (Supplementary Fig. 4a–e). However, more sensitive quantitative measurements revealed significant vestibular impairment. OVAR testing showed markedly reduced eye movement amplitudes across all tested frequencies in KO mice (Fig. 4f). Similarly, aVOR analysis demonstrated decreased gain values up to 3.2 Hz in *Acbd7* KO mice compared with WT controls (Fig. 4g). These physiological deficits specifically implicate dysfunction in both the utricular macula and horizontal semicircular canal crista.

Quantitative morphological analysis demonstrated significant axonal retraction in both utricular and saccular maculae, with comparatively milder changes in the crista ampullaris (Fig. 4h). Synaptic quantification uncovered substantial reductions in hair cell synaptic pair counts across all three vestibular organs in KO animals (Fig. 4i). These structural deficits were accompanied by progressive loss of VGNs by 9 months of age (Fig. 4j). The differential neurofilament pathology between otolith organs (utricle and saccule) and semicircular canal cristae may reflect either intrinsic differences in synaptic architecture between these subsystems or variable compensatory capacities among vestibular end organs.

As *Acbd7* is broadly expressed across all vestibular hair cells, we next investigated whether its loss affects subtype specification. Our analysis revealed that the expression patterns and distribution of *Agbl1*, *Kcnj16*, *Cdk15*, and *Bmp6* remained unchanged following *Acbd7* KO (Supplementary Fig. 4f–i), demonstrating that *Acbd7* is dispensable for the maintenance of vestibular hair cell subtype identity. This finding aligns with our cochlear observations, supporting the conclusion that *Acbd7* regulates hair cell function in inner ear sensory epithelia.

### Conditional KO of *Acbd7* impairs hearing function and synaptic integrity

**Fig. 5:**
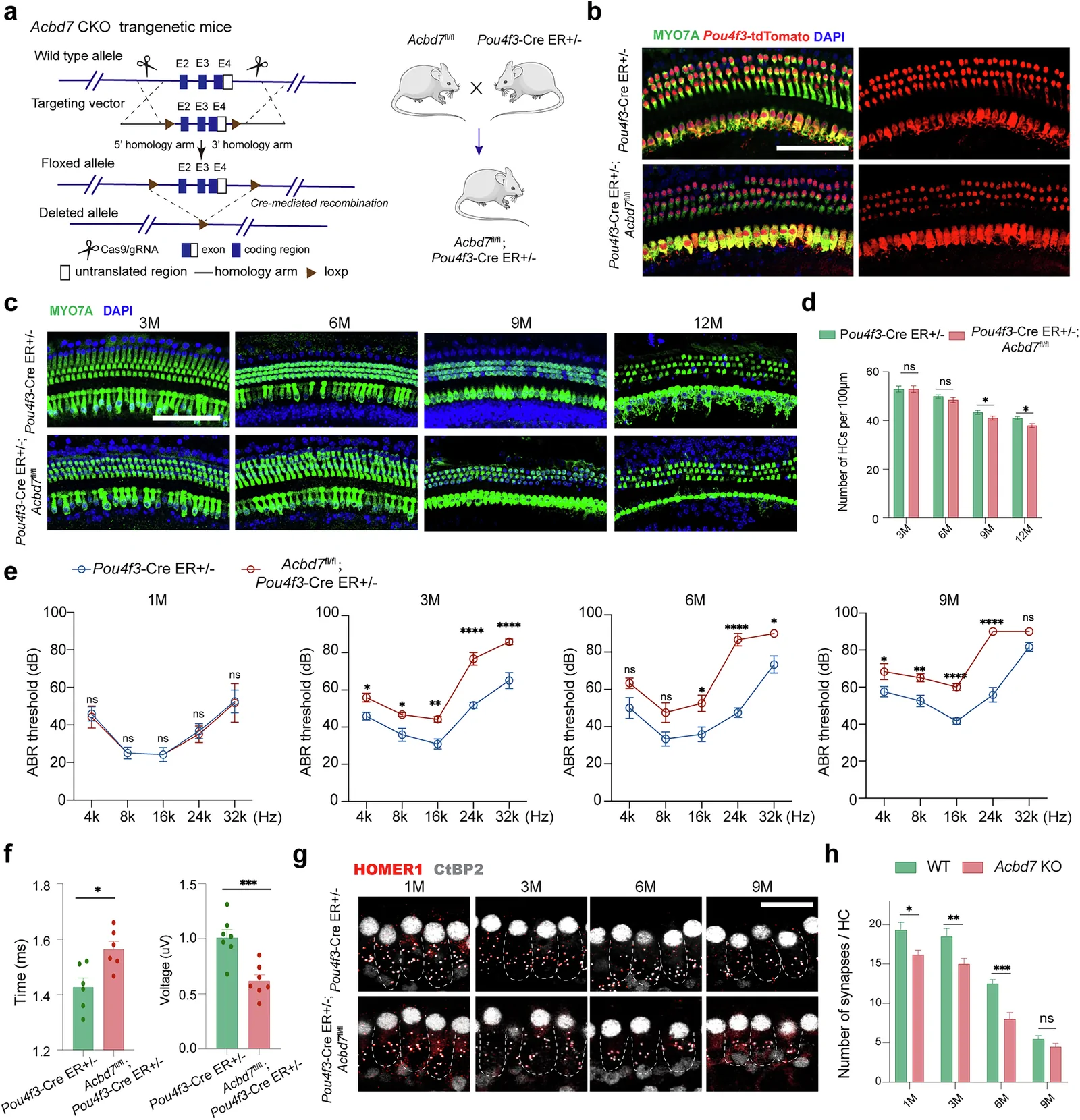
Hair cell‑specific *Acbd7* deletion leads to progressive hearing loss. **a**, Schematic of *Acbd7* conditional knockout (CKO) transgenic mouse model. Genetic strategy for *Acbd7* deletion in hair cells combined with the *Pou4f3*-Cre ER+/− system. **b**, Representative images showing Cre recombinase activity in hair cells from the middle turn of the cochlea. Cre recombinase driven by *Pou4f3* is labeled by tdTomato and hair cells are labeled with MYO7A (green). Scale bar, 50 μm. **c**, Representative images show MYO7A-labeled hair cells (green) from WT and *Acbd7* CKO mice in the middle turn of the cochlea. Scale bar, 50 μm. **d**, Quantification of MYO7A+ hair cell number (*n* = 12 mice per group) in the middle turn of the cochlea from *Pou4f3*-Cre ER and *Acbd7* CKO mice. **e**, ABR thresholds of *Pou4f3*-Cre ER and *Acbd7* CKO mice (*n* = 6 mice per group). **f**, ABR wave I latency and amplitude (*n* = 6 mice per group) of *Pou4f3*-Cre ER and *Acbd7* CKO mice (*n* = 6 mice per group). **g**, Synaptic impairment in *Acbd7* CKO mice in the middle turn of the cochlea. Presynaptic components were marked by CtBP2 (gray) and postsynaptic elements were marked by HOMER1 (red). Scale bar, 20 μm. **h**, Quantification of synapses in *Pou4f3*-Cre ER and *Acbd7* CKO IHCs at different ages (*n* = 6 mice per group). Unpaired Student’s *t* tests (two-tailed) were performed for part **f**. Two-way analysis of variance followed by Tukey’s multiple comparisons test was performed for parts **d**, **e**, and **h**. Data are presented as means ± SEMs. ****P < 0.0001, ***P < 0.001, **P < 0.01, *P < 0.05, n.s., not significant. ABR, auditory brainstem response; CKO, conditional knockout; DAPI, 4′,6-diamidino-2-phenylindole; gRNA, guide RNA; HC, hair cell.

To precisely investigate the hair cell-specific functions of *Acbd7* while eliminating potential confounding effects from systemic deletion, we generated an inducible conditional knockout (CKO) model by crossing *Acbd7*-floxed (*Acbd7*^fl/fl^) mice with *Pou4f3*-CreERT2 transgenic mice^54^ (Fig. 5a). Tamoxifen administration (75 mg/kg daily on postnatal days 2–4) efficiently induced Cre-mediated recombination in hair cells, as confirmed by tdTomato fluorescence under the control of *Pou4f3* promoter (Fig. 5b). Hair cell-specific deletion of *Acbd7* resulted in progressive OHC loss, with significant reductions observed in 9-month-old and 12-month-old *Acbd7*^fl/fl^ mice; *Pou4f3*-CreER+/− mice compared with *Pou4f3*-CreER+/− control mice (Fig. 5c,d and Supplementary Fig. 5a,b).

Auditory phenotyping demonstrated progressive, age-dependent hearing impairment in *Acbd7* CKO mice. At 1 month of age, ABR thresholds showed no significant differences between *Acbd7*^fl/fl^ mice; *Pou4f3*-CreER+/− mice and control (*Pou4f3*-CreER+/−) mice across all tested frequencies (Fig. 5e). However, by 3 months of age, *Acbd7* CKO mice developed significant hearing loss, particularly at high frequencies, which progressively worsened through 9 months of age. In addition, in *Acbd7* CKO mice, a reduction in the mean peak amplitude of ABR wave I and an elongation of latency were observed (Fig. 5f). Morphological examination confirmed synaptic degeneration, with *Acbd7* CKO mice exhibiting progressive loss of CtBP2/HOMER1-positive synaptic pairs (Fig. 5g,h). These results demonstrate that *Acbd7* expression is indispensable for hair cell synaptic maintenance and normal auditory function. The consistent synaptic pathology observed in both global KO and conditional KO models establishes *Acbd7* as a crucial molecular regulator of hair cell synaptic stability and function.

### *Acbd7* is essential for the hair cell calcium homeostasis

**Fig. 6:**
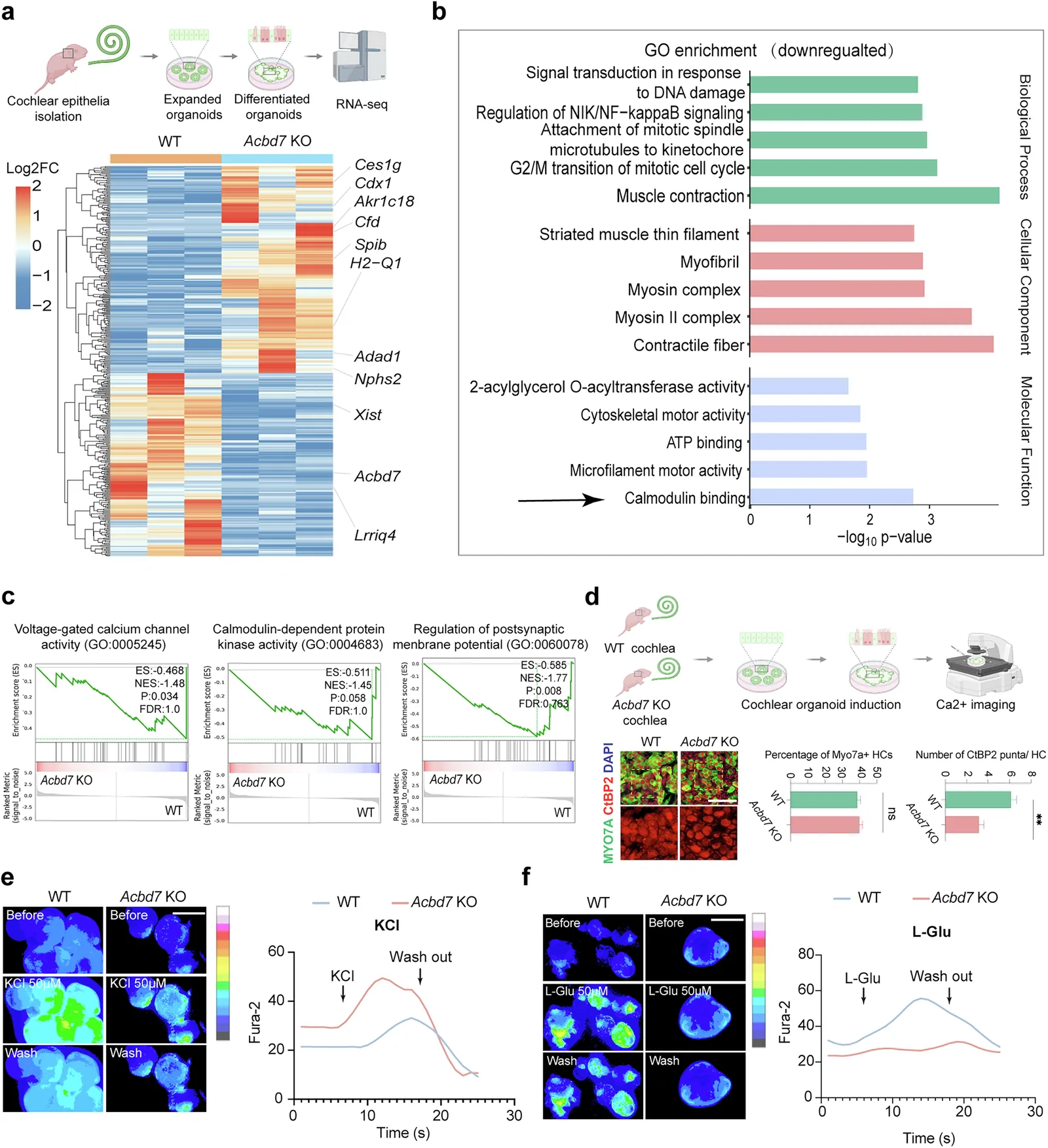
*Acbd7* regulates Ca^2+^ channel activity. **a**, Heatmap showing differentially expressed gene expression profile between *Acbd7* KO and WT cochleae. **b**, GO enrichment of downregulated differentially expressed genes upon *Acbd7* KO. **c**, Gene Set Enrichment Analysis enrichment of representative terms. **d**, Schematic depicting the mouse cochlear organoid culture and Ca^2+^ imaging. Immunofluorescence staining showing the percentage of MYO7A+ hair cells (green) and the number of CtBP2 (red) puncta per hair cell in WT and *Acbd7* KO mouse organoids. Scale bar, 20 μm (*n* = 8 organoids per group from three independent experiments). Unpaired Student’s *t* tests (two-tailed) were performed for part **d** and data are presented as means ± SEMs. **P< 0.01, n.s., not significant. **e**,**f**, Calcium imaging in mouse cochlear organoids in response to KCl and L-glutamate (L-GLU) stimulation. Scale bar, 100 μm (*n* = 6 organoids per group from three independent experiments). DAPI, 4′,6-diamidino-2-phenylindole; FC, fold change; GO, Gene Ontology; HC, hair cell; KO, knockout; NF, nuclear factor; WT, wild-type.

Cochlear IHCs and SGNs are functionally connected via synapses whose signaling depends on Ca^2+^-triggered release of glutamate from synaptic vesicles^55^. In OHCs, regulated Ca^2+^ influx is critical for survival and for the function of the electromotility protein PRESTIN^56,57^. To dissect how *Acbd7* influences hair cell function, we performed bulk RNA sequencing on cochlear epithelia from postnatal day 7 *Acbd7* KO and WT mice (Fig. 6a). Comparative transcriptomic analysis between *Acbd7 KO* and WT groups identified 200 upregulated and 177 downregulated DEGs with a standard fold change >1.5 and a *P*-value <0.05 (Fig. 6a). To gain further insights into the functions of *Acbd7*-regulated genes, downregulated DEGs were subjected to Gene Ontology (GO) enrichment analysis. The enriched GO terms in the biological process category were muscle contraction, microtubules to kinetochore, and so on (Fig. 6b). The GO terms enriched in the cellular component category were contractile fibers, myosin complexes, myofibrils, and so forth, suggesting the potential role of *Acbd7* in cytoskeletal regulation. Notably, molecular function analysis identified calmodulin binding, microfilament motor activity, and ATP binding as top enriched terms. These findings are particularly relevant as calmodulin-binding proteins have a key role in Ca^2+^ activity, serving as dynamic Ca^2+^ sensors that can respond to dynamic changes in Ca^2+^ concentrations^58^. Gene set enrichment analysis further revealed the enriched terms of the downregulated DEGs, including calmodulin-dependent protein kinase activity, voltage-gated calcium channel activity, and regulation of postsynaptic membrane potential (Fig. 6c). These results suggest that *Acbd7* may regulate Ca^2*+*^*-*related protein expression to influence hair cell function.

To validate the role of *Acbd7* in Ca^2*+*^ activity, we used an inner ear organoid model (Fig. 6d). Although *Acbd7* deletion did not affect hair cell number, we observed significant reductions in CtBP2 puncta (Fig. 6d), consistent with our in vivo synaptic phenotype. Calcium imaging using Fura-2 in mature organoids revealed diminished calcium responses in *Acbd7* KO organoids following stimulation with either 50 μM KCl or L-glutamate (Fig. 6e,f).

**Fig. 7:**
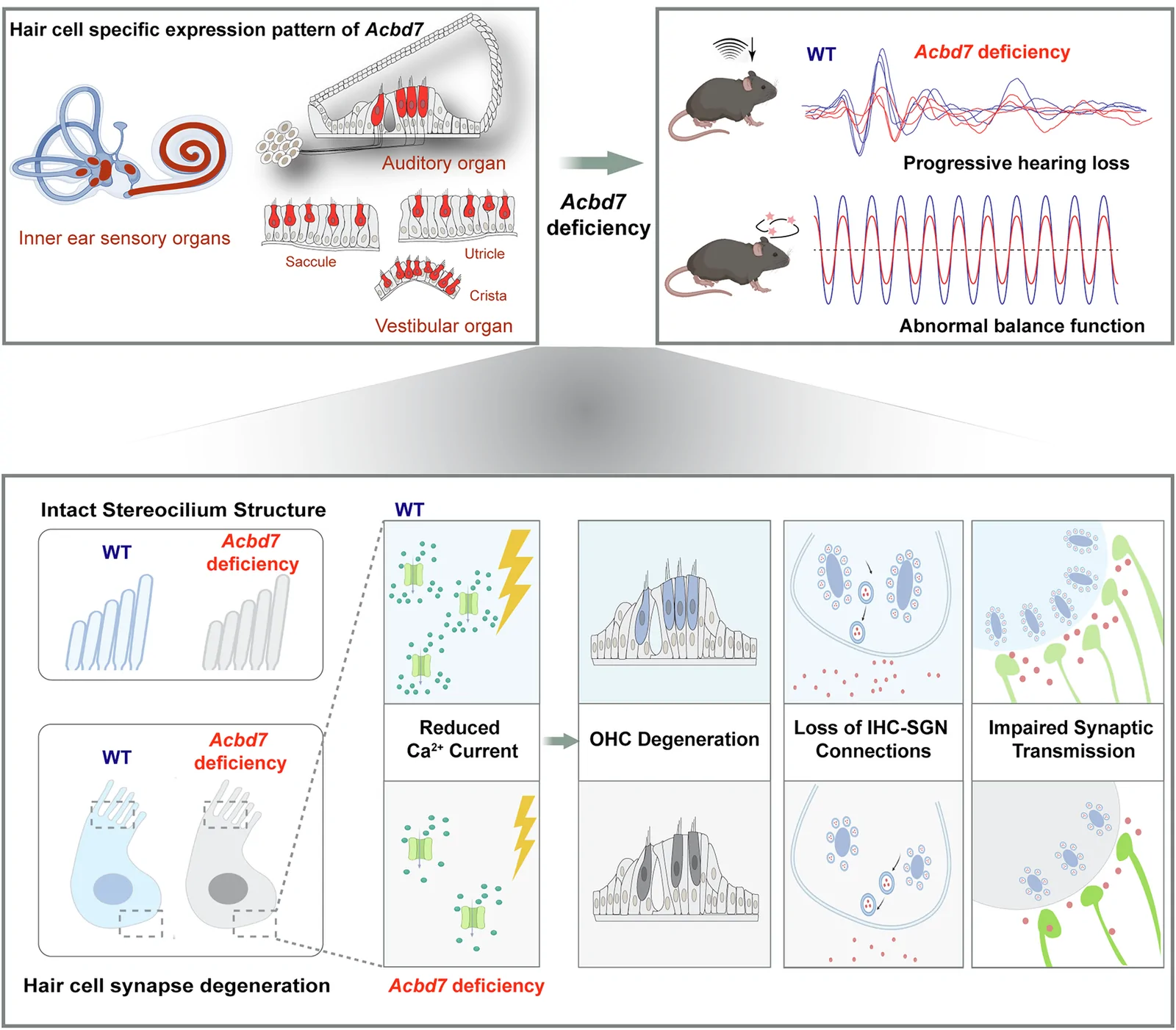
Maintenance of hair cell-dependent auditory and vestibular function critically requires *Acbd7.* *Acbd7* exhibits specific expression in inner ear hair cells, encompassing both cochlear and vestibular organs. Genetic ablation of *Acbd7* results in progressive auditory and vestibular dysfunction, primarily driven by impaired calcium signaling and consequent degeneration of cochlear hair cells and neuronal innervation. IHC, inner hair cell; OHC, outer hair cell; SGN, spiral ganglion neuron; WT, wild-type.

To elucidate how *Acbd7* influences hair cell function, we performed proteomic analysis of *Acbd7* KO cochleae. This analysis identified a set of differentially expressed proteins (Supplementary Fig. 5a,b) and revealed significant downregulation of pathways associated with fatty acyl-CoA binding and fatty acid metabolism (Supplementary Fig. 5c,d). The plasma membrane lipid bilayer is composed of phosphatidylcholine, cholesterol, and asymmetrically distributed phosphatidyl serine^59^. Fatty acid metabolism contributes to membrane excitability and synaptic transmission by modulating ion channel activity and maintaining lipid asymmetry^60,61^. As a member of the acyl‑CoA‑binding protein (ACBP) family, whose role in fatty acid metabolism is well established^62,63^, *Acbd7* may modulate fatty acid metabolism and contribute to the intracellular calcium homeostasis, thereby preserving functional integrity in both IHCs and OHCs (Fig. 7).

## Discussion

We have identified *Acbd7* as a molecular marker specifically expressed in inner ear hair cells. To investigate the progressive hearing loss observed in *Acbd7*-deficient mice, we performed comprehensive functional and morphological analyses. Our results reveal that the profound auditory deficits in *Acbd7* KO mice arise from dysfunction in both IHCs and OHCs. Notably, although IHCs in *Acbd7* KO mice maintained normal stereociliary morphology, they exhibited substantial synaptic dysfunction and structural disruption at ribbon synapses. Electrophysiological recordings further confirmed comparable MET channel activity in IHCs between WT and *Acbd7* KO mice. Concurrently, we observed functional impairment and progressive loss of OHCs, indicating that *Acbd7* is essential for maintaining the function and integrity of both IHCs and OHCs. Collectively, these results rule out impaired MET channel activity as a primary cause of hearing impairment in *Acbd7* KO mice and instead demonstrate that the auditory dysfunction arises primarily from defective IHC synaptic transmission and OHC loss. Furthermore, our findings demonstrate that *Acbd7* is essential for synaptic maintenance in vestibular hair cells as well. Taken together, this work identifies *Acbd7* as an essential regulator for both auditory and vestibular function in the inner ear.

Despite emerging evidence suggesting a role of *Acbd7* in neurobiology, its function in the inner ear has remained largely unexplored. Our study provides direct genetic and functional evidence defining its essential role in auditory and vestibular systems. In the mouse hypothalamus, the ACBD7 protein is produced by GABAergic neurons and processed into a bioactive peptide referred to as nonadecaneuropeptide in response to catabolic signals^42^, indicating the role of the ACBD7 protein in neurological function. Previously, no evidence addressed whether or how *Acbd7* affects neurons or other cell types, owing to a lack of direct gene-level or protein-level intervention. By generating and characterizing both *Acbd7* KO and CKO mice, we provide definitive genetic evidence that *Acbd7* is indispensable for auditory and vestibular function. Although our study establishes its critical role in peripheral sensory systems, the potential functions of *Acbd7* in the central nervous system remain to be elucidated.

The observed disparity between severe auditory deficits and relatively preserved vestibular function in *Acbd7* KO mice may reflect compensatory mechanisms in the balance system, which integrates multiple sensory inputs including visual and proprioceptive cues^64–67^. Thus, it is not surprising that, as the vestibular reflex involves complex projections, including vestibulo-ocular and vestibulo-spinal reflexes^68^, the complex interaction might compensate for the symptoms caused by *Acbd7* deficiency. However, the remarkable abnormalities in OVAR and aVOR tests suggest that *Acbd7* deficiency could underlie certain vertigo conditions, warranting investigation of its potential role in human delayed-onset hearing loss and balance disorders. The progressive hearing loss observed in *Acbd7*-deficient mice underscores its essential role in auditory maintenance and highlights the clinical importance of investigating its relevance in human hearing function. To this end, future studies incorporating human cochlear expression profiles and functional analyses will be critical for defining its specific role in human hearing function.

Evidence from Ca^2+^ imaging, electrophysiological recordings, and transcriptomic analyses indicates that disruption of calcium signaling is central to the functional defects in *Acbd7* KO hair cells. Calcium homeostasis in hair cells is tightly regulated by membrane ion channels and EF‑hand proteins, including PVALB, CALB1, CALB2, and oncomodulin, which buffer Ca^2+^ transients and modulate intracellular Ca^2+^ signaling^69,70^. The deficiency of these proteins leads to abnormal vesicle release in hair cells and delayed maturation of afferent synapses^24,57,71^. We found that *Acbd7* deficiency leads to reduced PVALB expression, further supporting the link between *Acbd7* and intracellular calcium regulation in hair cells. However, how Acbd7 regulates PVALB expression and calcium binding in hair cells requires further investigation through molecular mechanism study. In addition, proteomic analysis revealed that *Acbd7* deficiency disrupts fatty acid metabolic pathways. Studies have shown that fatty acid metabolism contributes to Ca^2+^ homeostasis^72,73^, thus establishing a clear link between these two processes. In hair cells, we demonstrated that *Acbd7*, which regulates fatty acid metabolism, is linked to intracellular calcium activity, thereby affecting hair cell function and survival. However, further investigation is needed to clarify the specific fatty acid pathways and calcium channels through which this regulation occurs.

In conclusion, this study establishes *Acbd7* as a pivotal regulator of hair cell function. The *Acbd7*-deficient phenotype faithfully recapitulates the hallmark pathology observed in inner ear disorders, offering both a robust experimental model for therapeutic development in auditory–vestibular disorders and fundamental insights into the critical interplay between lipid metabolism and calcium signaling in sensory neurotransmission. These discoveries reshape our understanding of inner ear dysfunction mechanisms while providing transformative opportunities for developing targeted interventions against hearing and balance disorders.

## Supplementary information

## Data Availability

Bulk RNA-sequencing data in this study are available through the National Center for Biotechnology Information Gene Expression Omnibus (NCBI SRA) (PRJNA1148216).
