# Supplementary material for: Acbd7 is essential for preserving hair cell-mediated auditory and vestibular function
Source: Exp Mol Med. 2026 Aug 14;58(8):2792–806. doi: 10.1038/s12276-026-01812-1 (PMC13538670; doi:10.1038/s12276-026-01812-1)
Supplement: Supplementary file 1 [file 12276_2026_1812_MOESM1_ESM.pdf]

Supplementary Information

Acbd7 is essential for preserving hair cell-mediated auditory and vestibular function

Mingxuan Wu[1,2,3,4,#], Gaogan Jia[1,2,3,4,#], Yanyan Jia[1,2,3,4,5,#], Yikang Huang[1,2,3,4], Yunjie Li[1,2,3,4], Yaoqian Liu[1,2,3,4], Yiyun Lou[1,2,3,4], Tianshi Sun[6], Xintong Qiu[1,2,3,4], Mingyu Xia[1,2,3,4,*], Huawei Li[1,2,3,4,5,*], and Wenyan Li[1,2,3,4,5,*]

Supplementary Figures and Figure Legends

Supplementary Fig. 1

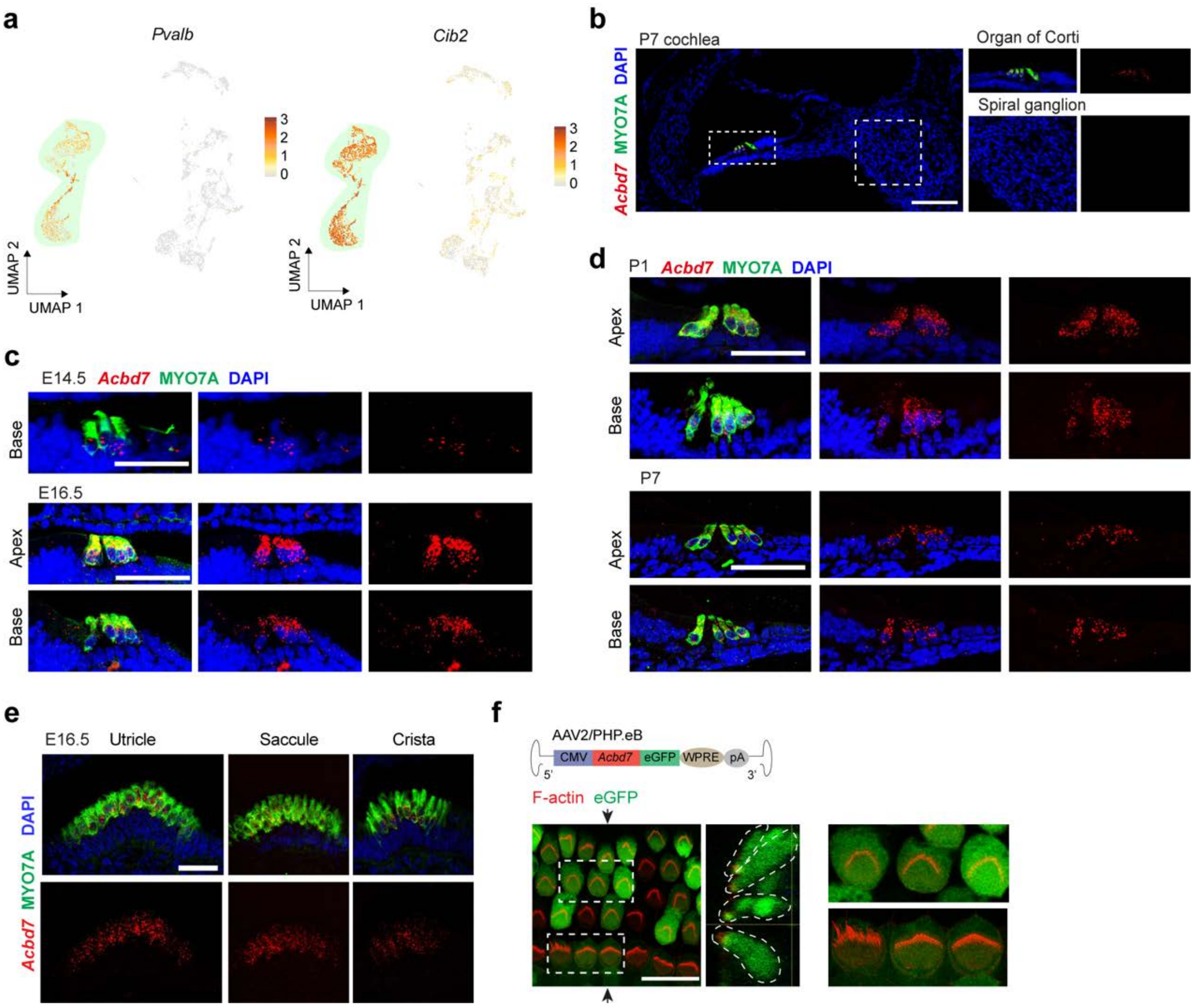

Supplementary Fig. 1 Expression of *Acbd7* in cochlea and vestibular organs.

a Feature plots of hair cell markers *Pvalb* and *Cib2*. **b** RNAscope detection of *Acbd7* (red) in the cochlea. Hair cells were labeled with MYO7A (green). Scale bar, 100 µm.

c Expression pattern of *Acbd7* (red) in developing cochlea of embryonic stages. Hair cells were labeled with MYO7A (green). Scale bar, 50 μm. **d** Expression pattern of *Acbd7* (red) in P1 mouse cochlea. Scale bar, 50 μm. **e** Expression pattern of *Acbd7* (red) in E16.5 mouse vestibular organs. Scale bar, 50 μm. **f** Generation of *Acbd7*-overexpressing adeno-associated virus (AAV) and subcellular localization analysis. The AAV was constructed based on the AAV PHP.eB vector and carries the following components: a CMV promoter driving expression of the full-length murine *Acbd7* coding sequence, followed by an eGFP reporter, a WPRE (Woodchuck Hepatitis Virus Post-Transcriptional Regulatory Element), and a polyadenylation (PolyA) signal.

Supplementary Fig. 2

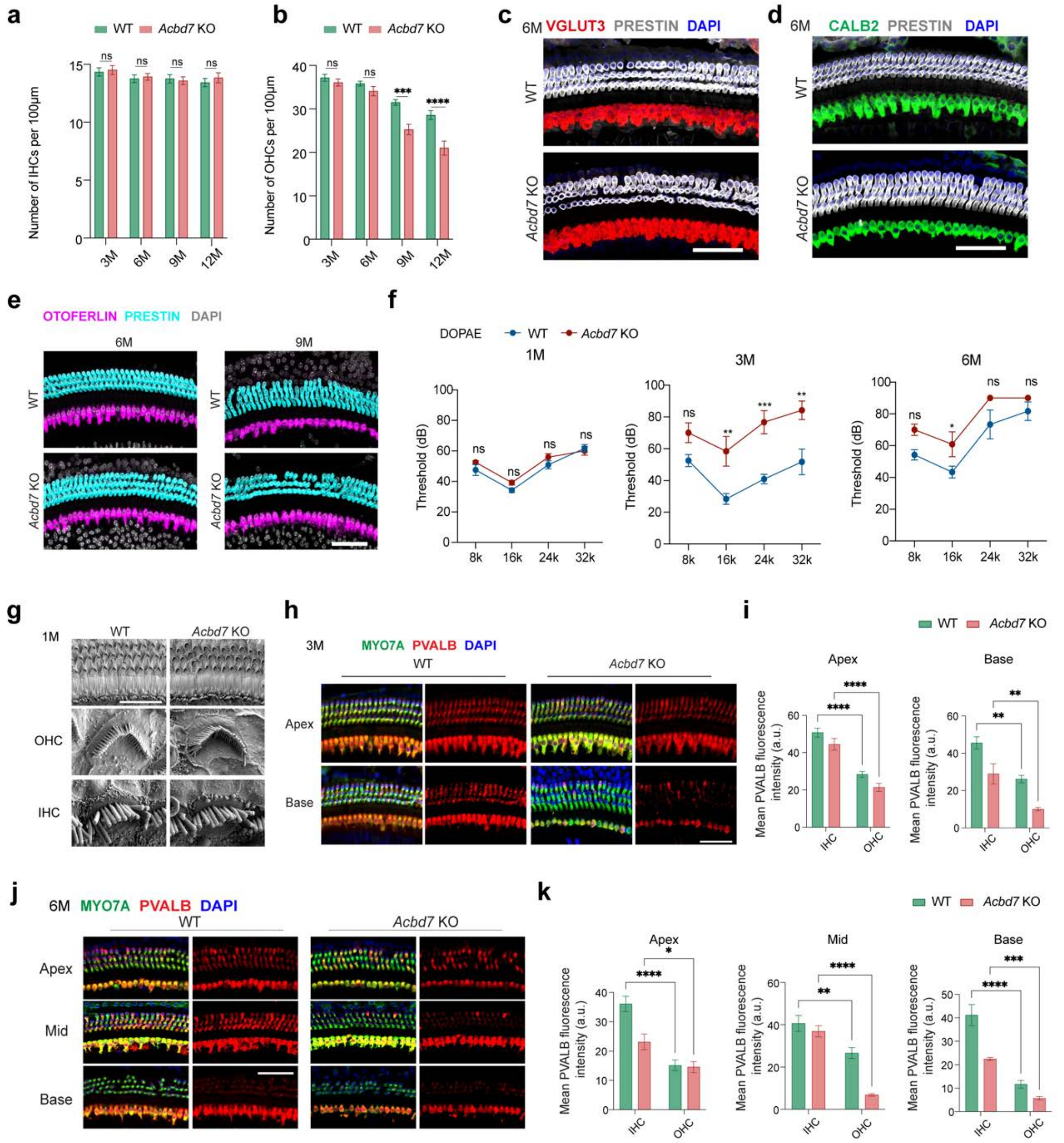

Supplementary Fig. 2 Immunofluorescence analysis of cochlear hair cell markers in WT and *Acbd7* KO Mice.

a, **b** Quantification of inner (**a**) and outer (**b**) hair cell numbers, identified by MYO7A labeling and location, in the middle turn of the cochlea from WT and *Acbd7* KO mice (n = 12 mice per group). **c** and **d** Immunofluorescence staining of IHC and OHC markers at 6 months of age. IHCs were labeled with VGLUT3 (red) and CALB2 (green); OHCs labeled with PRESTIN (gray). Images were acquired from the middle turn of the cochlea. Scale bar, 50 μm. **e** Immunostaining of OTOFERLIN (magenta) in IHCs and PRESTIN (cyan) in OHCs in WT and *Acbd7* KO mice at 6 and 9 months of age, imaged from the middle turn of the cochlea. Scale bar, 50 μm. **f** DPOAE measurement of WT and *Acbd7* KO mice. (n=6 mice per group). **g** Morphological assessment of OHCs and IHCs by SEM in 1-month-old WT and *Acbd7* KO mice,

acquired from the middle turn of the cochlea. Scale bar, 20 µm. **h** Immunofluorescence staining of PVALB (red) and MYO7A (green) staining in cochlear hair cells from 3-month-old WT and *Acbd7* KO mice. Images were acquired from the apical and basal turns of the cochlea. Scale bar, 50 µm. **i** Quantification of mean PVALB fluorescence intensity of the apical and basal turn of the cochlea from 3-month-old WT and *Acbd7* KO mice (n=6 mice per group). **j** PVALB expression (red) in cochlear hair cells from 6M WT and *Acbd7* KO mice. Hair cells are were labeled with MYO7A (green). Scale bar, 50 µm. **k** Quantification of mean PVALB fluorescence intensity in IHCs and OHCs from 6-month-old WT and *Acbd7* KO mice (n=6 mice per group). Two-way ANOVA followed by Tukey's multiple comparisons test was performed for **a, b**, **f**, **i**, and **k**. Data are presented as means ± SEMs. ****$p < 0.0001$, ***$p < 0.001$, **$p < 0.01$, *$p < 0.05$, n.s., not significant.

Supplementary Fig. 3

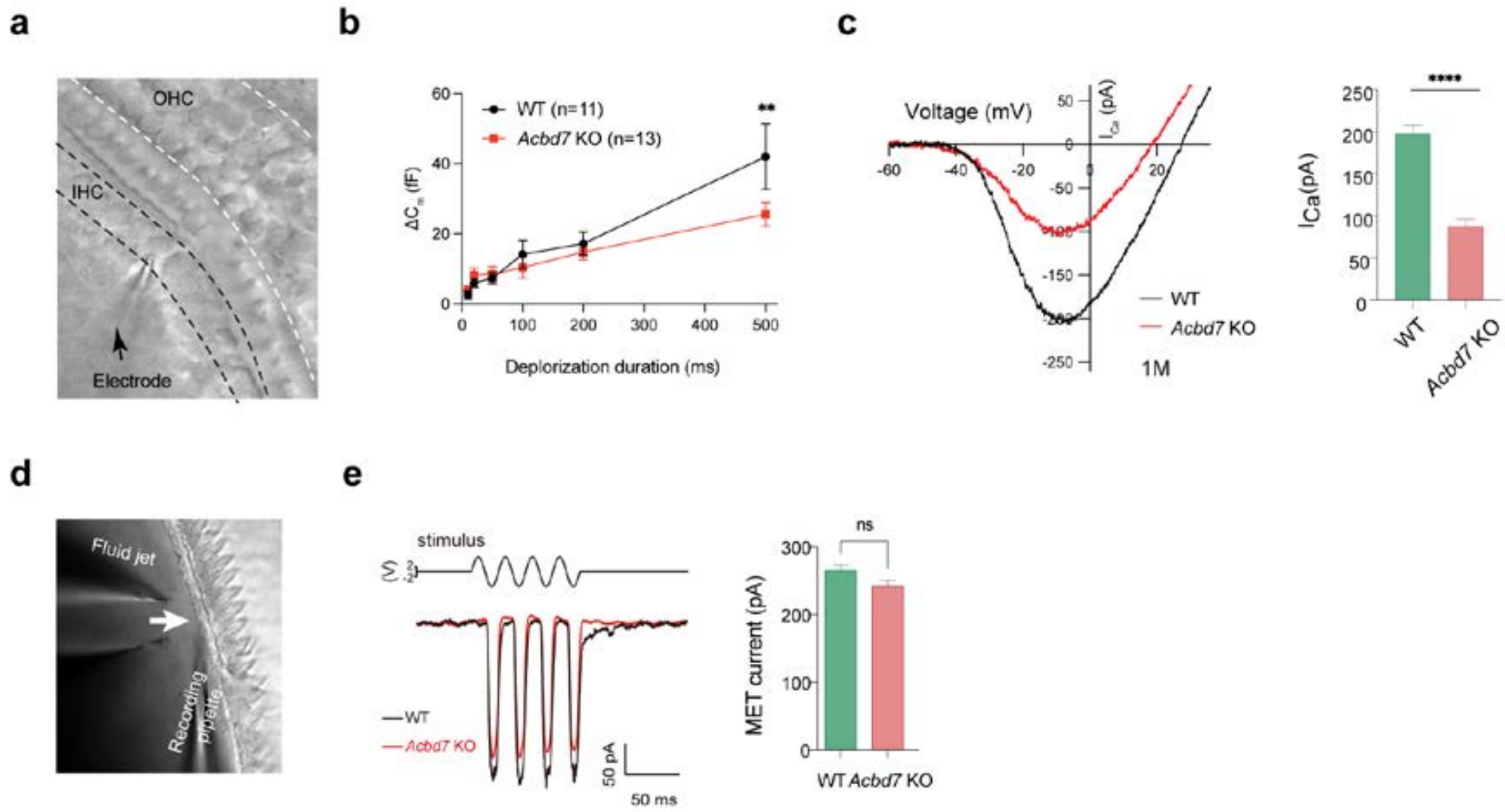

Supplementary Fig. 3 Functional analysis of *Acbd7* KO hair cells.

a The bright field image of electrophysiological examination. The electrode is indicated by the arrow. **b** Changes in IHC membrane capacitance (△Cm) in response to depolarization in 1-month-old WT (n=11 cells from 3 mice) and *Acbd7* KO (n=13 cells from 3 mice) mice from the apical turn. **c** Representative Ca^{2+} current traces of IHCs from WT (black) and *Acbd7* KO (red) mice are shown on the left. Recordings were obtained from apical cochlear turns of 1-month-old mice (n=10 cells per group from 3 mice). Quantified pooled data from independent cells are presented on the right. **d** Configuration of recording pipettes positioned around a single IHC in MET current detection. **e** Representative traces of MET responses recorded from apical IHCs of 1-month-old WT (black) and *Acbd7* KO (red) mice. Right panel shows quantification of peak MET current amplitudes from WT (n=13 cells) and *Acbd7* KO (n=11 cells). Unpaired Student's *t*-tests (two-tailed) were performed for **b**, **c** and **e**. Data are presented as means ± SEMs. ****$p < 0.0001$, ***$p < 0.001$, **$p < 0.01$, *$p < 0.05$, n.s., not significant.

Supplementary Fig. 4

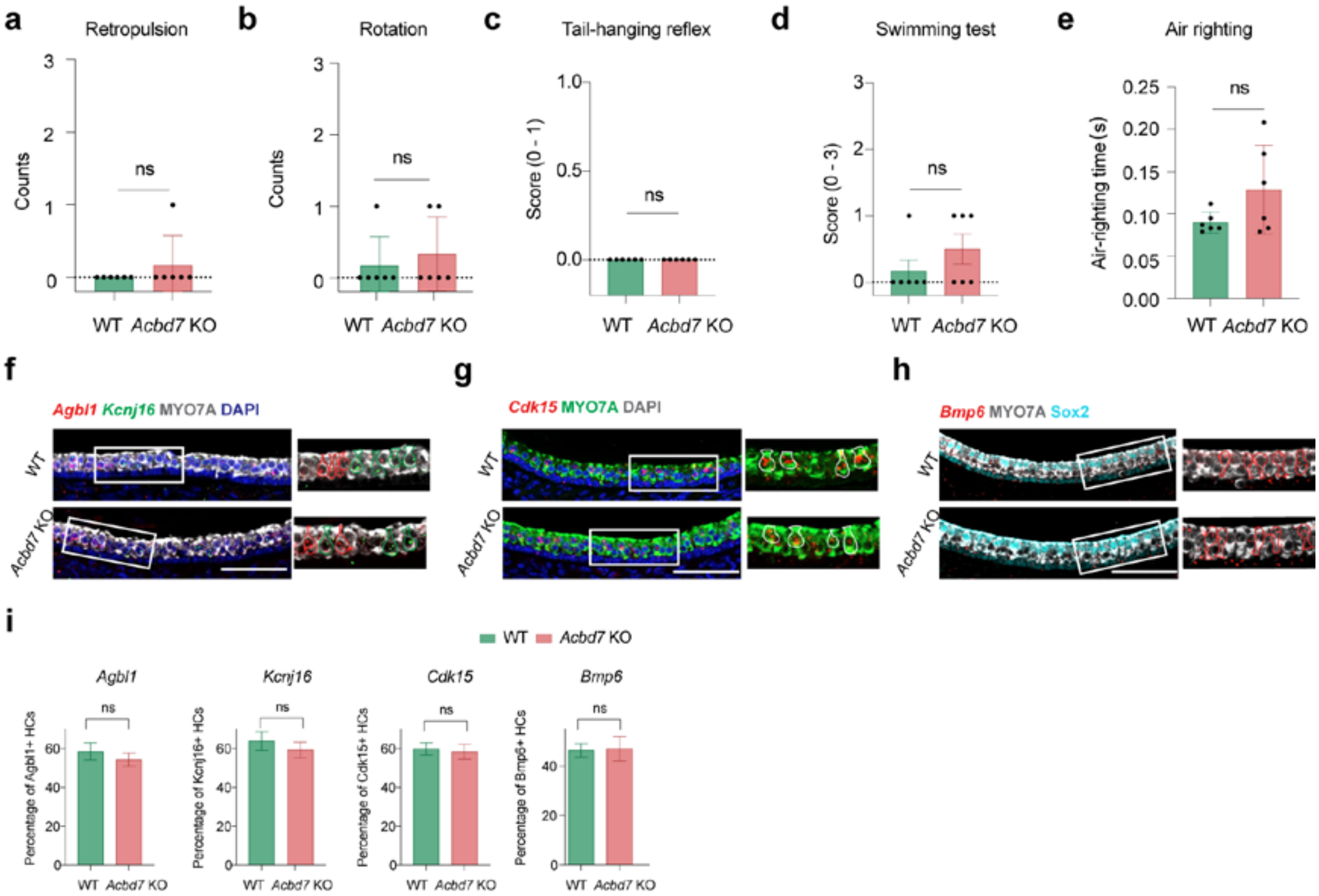

Supplementary Fig. 4 Vestibular behavioral tests of WT and *Acbd7* KO mice.

a-e Retropulsion Test (**a**), Rotation Test (**b**), Tail-Hanging Reflex Test (**c**), Swimming Test (**d**), and Air Righting Reflex Test (**e**) of 9-month-old WT and *Acbd7* KO mice. (n=6 mice per group). **f-h**, Expression of vestibular hair cell subtype markers in the utricle of 3-month-old WT and *Acbd7* KO mice. Hair cells were labeled by MYO7A. Scale bar, 50 µm **f** *Agbl1* (red) and *Kcnj16* (green). **g** *Cdk15* (red) **h** *Bmp6* (red). Type II hair cells are identified by co-expression of MYO7A and SOX2 (cyan). **i** Proportions of *Agbl1+*, *Kcnj16+*, *Cdk16+ and Bmp6+* hair cells in the utricle of 3-month-old WT and *Acbd7* KO mice (n=6 mice per group). Unpaired Student's *t*-tests (two-tailed) were performed for **a-e** and **i**. Data are presented as means ±SEMs. n.s., not significant.

Supplementary Fig. 5

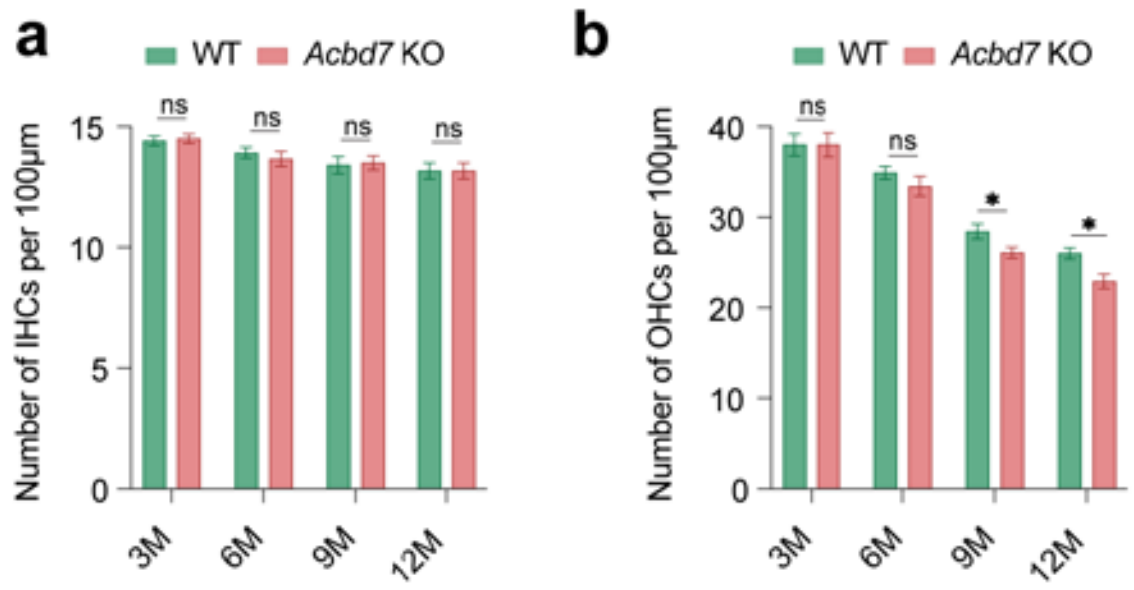

Supplementary Fig. 5 Hair cell-specific deletion of Acbd7 results in OHC loss.

a Quantification of IHC number in the cochlea from *Pou4f3*-Cre ER and *Acbd7* CKO mice, analyzed from the middle turn of the cochlea (n=12 mice per group) identified by MYO7A labeling. **b** Quantification of OHC number in the cochlea from *Pou4f3*-Cre ER and *Acbd7* CKO mice, analyzed from the middle turn of the cochlea (n=12 mice per group) identified by MYO7A labeling. Two-way *ANOVA* followed by Tukey's multiple comparisons test was performed for **a** and **b**. Data are presented as means ±SEMs. *$p < 0.05$, n.s., not significant.

Supplementary Fig. 6

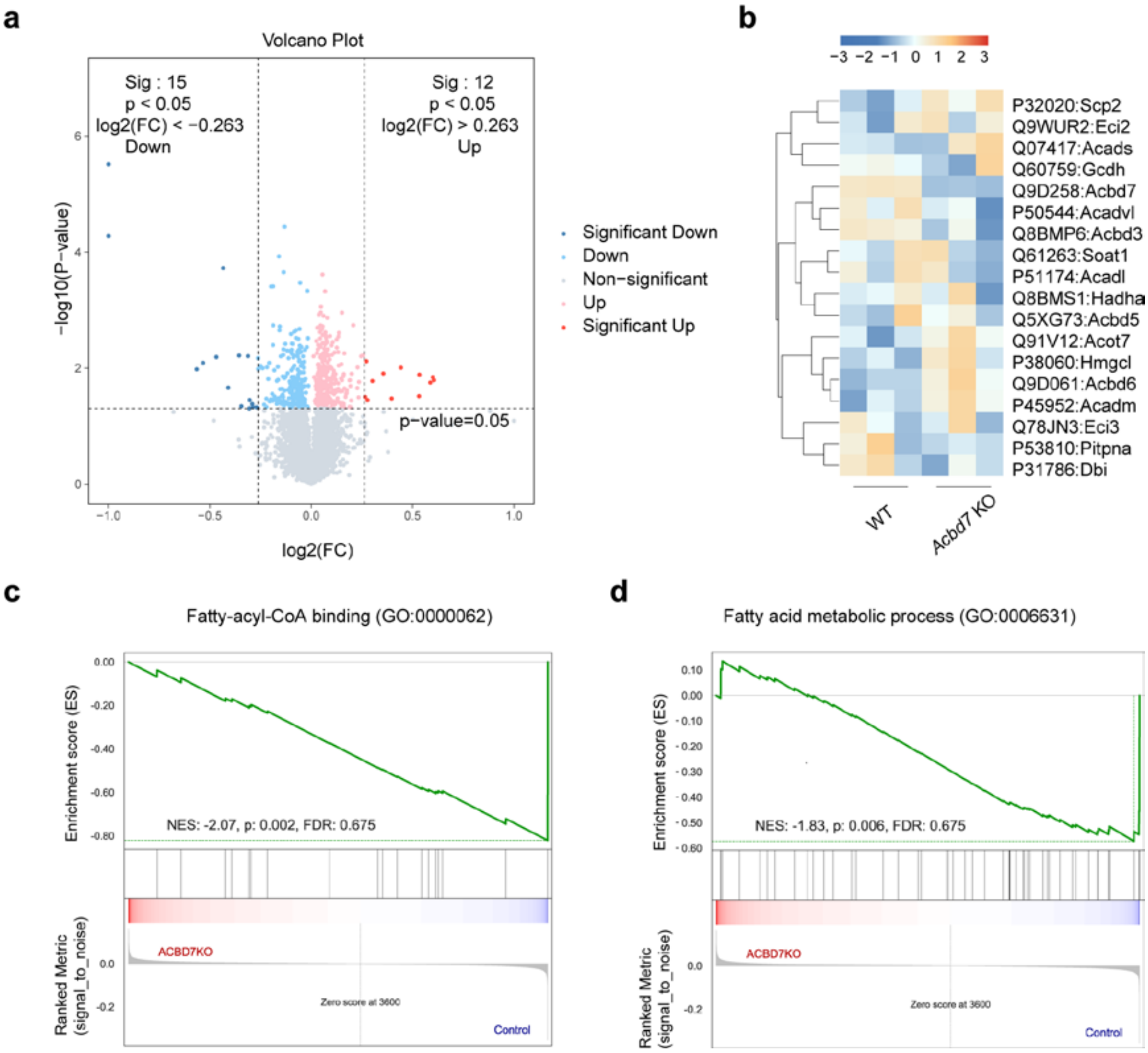

Supplementary Fig. 6 Functional proteomic analysis of *Acbd7* KO cochleae.

a Volcano plot of differentially expressed proteins in WT and *Acbd7* KO mouse cochleae harvested in vivo at P7. **b** Heatmap showing differentially expressed proteins in fatty acyl-CoA binding and fatty acid metabolism pathway in WT and *Acbd7* KO cochleae. **c** and **d** GO term analysis of downregulated protein and GSEA of DEGs from WT and *Acbd7* KO mice cochlea.

Supplementary Table 1. Primers for genotyping.

Gene	Primer
Acbd7 **KO Wild type Forward**	5′-CCGTTGGCATGTCCTTGCTAT-3′
Acbd7 **KO Wild type Reverse**	5′-CTTCCCATTTGGCCTTACCCT-3′
Acbd7 **KO Mutant Forward**	5′-GGGCTTTATGAAGCTCATGTGTGG-3′
Acbd7 **KO Mutant Reverse**	5′-GAGCCAAGTCTACCTGTCACTCCATA-3′
Pou4f3 **cre ER Common**	5′-GTGGGGGAGAGGGGAGGCAG-3′
Pou4f3 **cre ER Wild type reverse**	5′-GAGAGAGCGCGGGGGAGACA-3′
Pou4f3 **cre ER Mutant reverse**	5′-CGGTTCACCAGGGTGTCGCC-3′
Acbd7 $^{\mathbf{flox(exon2\text{-}4)}}$ **Forward**	5′-AGTGCTACTGGTCCAATCAAAGAAG-3′
Acbd7 $^{\mathbf{flox(exon2\text{-}4)}}$ **Reverse**	5′-AAGACCTCAGTGATTGGCTGGAACT-3′
***Rosa26*-tdTomato WT Forward**	5′- AAG GGA GCT GCA GTG GAG TA-3′
***Rosa26*-tdTomato WT Reverse**	5′- CCG AAA ATC TGT GGG AAG TC-3′
***Rosa26*-tdTomato Mutant Forward**	5′- GGC ATT AAA GCA GCG TAT CC-3′
***Rosa26*-tdTomato Mutant Reverse**	5′- CTG TTC CTG TAC GGC ATG G-3′

Supplementary Table 2. List of primary antibodies.

Antibodies	Source	Identifier or Catalog Number
Mouse anti-CtBP2	BD Transduction	612044
Mouse anti- Otoferlin	Abcam	ab53233
Rabbit anti-MYO7A	Proteus BioSciences	25-6790
Rabbit anti-Vglut3	Synaptic Systems	135203
Goat anti-SOX2	R&D System	AF2018
Goat anti-Prestin	Santa Cruz Biotechnology	SC-22692
Rabbit anti-GAPDH	Abcam	ab8245
Rabbit anti-SOX9	Abcam	ab185966
Rabbit anti-Homer1	Synaptic Systems	160002
Mouse anti-TUJ1	Biolegend	801202
Rabbit anti-MAP2	Cell Signaling Technology	4542
Rabbit anti-CALB2	Abcam	ab254070
Chicken anti-NF	Abcam	ab4680
Mouse anti-PSD95	Invitrogen	MA1-045
Mouse anti-Parvalbumin	Sigma-Aldrich	P3088
